# Progress in cancer drug delivery based on AS1411 oriented nanomaterials

**DOI:** 10.1186/s12951-022-01240-z

**Published:** 2022-01-31

**Authors:** Xin Tong, Lu Ga, Jun Ai, Yong Wang

**Affiliations:** 1 College of Chemistry and Environmental Science, College of Geographical Science, Inner Mongolia Key Laboratory of Environmental Chemistry, Inner Mongolia Normal University, 81 Zhaowudalu, Hohhot, 010022 China; 2 College of Pharmacy, Inner Mongolia Medical University, Jinchuankaifaqu, Hohhot, 010110 China

**Keywords:** AS1411, Cancer treatment, Nanomaterials, Analytical imaging, Targeted drug delivery

## Abstract

Targeted cancer therapy has become one of the most important medical methods because of the spreading and metastatic nature of cancer. Based on the introduction of AS1411 and its four-chain structure, this paper reviews the research progress in cancer detection and drug delivery systems by modifying AS1411 aptamers based on graphene, mesoporous silica, silver and gold. The application of AS1411 in cancer treatment and drug delivery and the use of AS1411 as a targeting agent for the detection of cancer markers such as nucleoli were summarized from three aspects of active targeting, passive targeting and targeted nucleic acid apharmers. Although AS1411 has been withdrawn from clinical trials, the research surrounding its structural optimization is still very popular. Further progress has been made in the modification of nanoparticles loaded with TCM extracts by AS1411.

**Graphical Abstract:**

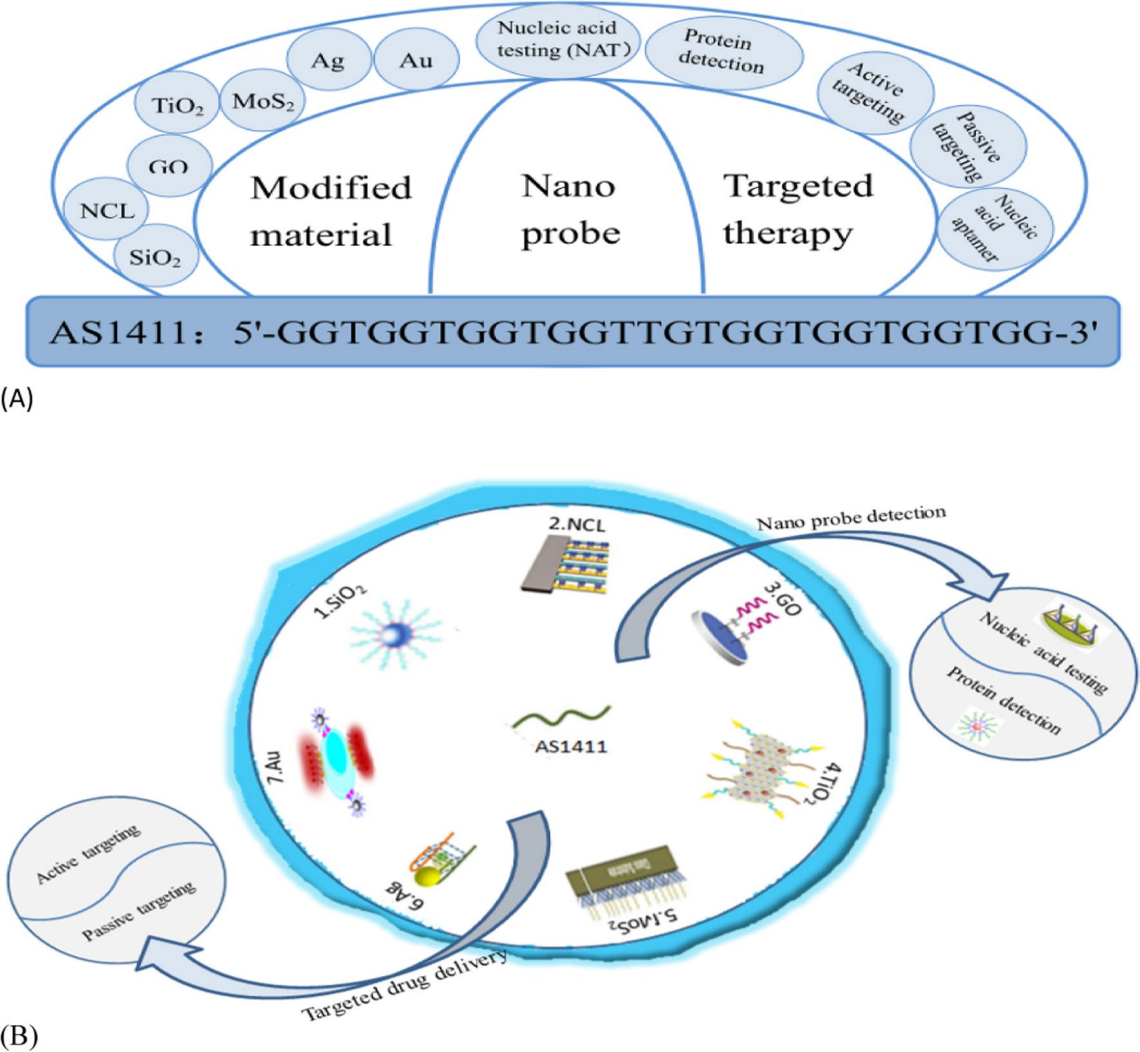

## Introduction

Cancer is a kind of disease caused by abnormal cell proliferation in the body. Such proliferation may invade or spread to other parts of the body, and these cells gather to form malignant tumors, which is one of the serious diseases threatening human health and life [1, 2]. Cancer, also known as malignancy, It is a kind of malignant growth of cells caused by gene mutation, resulting in abnormal cell differentiation, proliferation and malignant growth, which can proliferate indefinitely and have the ability to metastasize tumors, which can cause infectious diseases in surrounding tissues and organs. Malignant tumor cells usually show symptoms such as lack of appropriate signals to regulate proliferation and differentiation, endless proliferation and differentiation, programmed cell death and failure, unlimited cell division, accelerated angiogenesis, normal tissue invasion and metastasis formation, etc. These abnormal cells occur when human cells undergo genetic mutations that are influenced by carcinogens [3–7].

The growth rate of cancer cells is much faster than that of normal cells. In the process of its infinite proliferation and diffusion, a large amount of food nutrition, the body will secrete toxins, and the ability of cancer cells to metastasize, the infection of surrounding normal tissues and organs, even through the blood system and lymphatic system to the whole body [7]. At present, at the time of clinical diagnosis, most patients' malignant tumor cells have invaded and spread to normal tissues. Chemotherapy is the only feasible method to kill the diseased cells and prolong the life of patients [8–12]. In the past century, the incidence and mortality of cancer have been increasing continuously, and it has become one of the most important causes threatening human health [13–18].

Based on the biological aptamers of nanomaterials, this paper discusses the combination of AS1411 aptamers with various new nanomaterials to generate different nanocomposites. Nanomaterials discussed in this paper mainly include graphene nanomaterials, mesoporous silica nanomaterials, silver nanomaterials, gold nanomaterials and other nanomaterials. These generated nucleic acid functionalized nanomaterials have a wide range of applications. This review highlights recent research using the AS1411 aptamer-nanomaterial hybrid platform in biosensors, biomedics, drug targeted delivery, and nanoprobe imaging.

## Tumor markers

Cancer markers are kind of biological molecules with indication, they are produced in tumor cells, or by the tumor cells induced by tumor cells. Tumor markers include RNA [19–23], enzymes [24–28], DNA [29–31], etc. Due to the obvious difference in the expression level between tumor cells and normal cells, it has been used as an important basis for cancer diagnosis [29–31]. A 2012 review by He Xin et al showed that Nucleolin (C23) is an evolutionarily conserved nucleolus-cytoplasmic membrane shuttle protein, one of the most abundant proteins in eukaryotic nucleoli. Nucleolar protein is the most abundant phosphorylated protein in the nucleus of normal cells and has a variety of biological functions [32]: Regulation of biosynthesis of ribosomes and mature, involved in cell proliferation, growth, embryogenesis, cytokinesis, copy, the happening of the nucleolus chromatin and the function of the resistance to apoptosis, ligands can be from the cell surface to the nucleus, can also be in metastatic and excessive expression in the cytoplasm of rapidly dividing cells and transferred to the cell membrane [33–36].

## DNA rich in G bases

The tertiary structure of DNA has been found in nature. These guanine-rich structures are present in the telomere and promoter regions [37]. The guanine-rich sequences form various G-tetrastrands [38]. G-rich oligonucleotide molecules are somewhat different from others in that they are more likely to fold into G-quadruplex structures under physiological conditions than other nucleic acids that do not contain G-sequences. Nucleotide sequences rich in G bases have a certain anti-proliferation effect in tumor cells.

### G-quadruplex structure

G-quadruplex (G4) was first discovered by Gellert et al. In 1962, G-quadruplex is composed of DNA or RNA strands rich in serially repeated guanine (G) [39] induced by metal ions, and under certain ionic strength and appropriate pH conditions, It is a special secondary structure of nucleic acid formed by the accumulation of tetrad structure formed by Hoogsteen hydrogen bond [40], and it is also the supra-molecular structure of G-rich nucleic acid [41–44]. Advances in the chemical and structural biology of G-quadruplex have enabled the design of specific G4 aptamers to be used as novel anticancer agents and suitable for clinical trials [45]. As therapeutic agents, G-rich aptamers may have some advantages over monoclonal antibodies and other oligonucleotide based approaches. For example, tetra-stranded oligonucleotides are non-immunogenic, heat stable, and have increased resistance to serum nucleases and enhanced cell uptake compared to non-structural sequences [46–48]. It should be said that the G4 in AS1411 derivatives forms nuclear bases and main chains with substituting sequences, and the substitution of these nuclear bases and main chains can improve the chemical and biological properties of AS1411 [49]. Nucleic acids rich in tandem repeat G form Hoogsteen hydrogen bonds between corresponding G bases, so that 4 or 4 G-rich nucleic acid fragments are aggregated to form a G4 structure, whose basic structural unit is G-tetrad [50–52]. Through the longitudinal hydrophobic π-π interaction, the tetrad plane is stacked layer by layer and formed by Loop connection to form G4 [53]. G4 has a complex topological structure, which can be formed by one, two or more G-rich nucleic acids into intra-molecular or intermolecular quadruplex, which can be divided into parallel and anti-parallel structural forms according to the main chain (G-chain) direction of G4 [54]. The configuration of G4 is strongly dependent on the type of cations, and the G4 structure is further stabilized by the order of monovalent cations (K^+^ > Na^+^ > NH_4_^+^ > Li^+^), mainly Na^+^ and K^+^ [55, 56], especially in the solution containing K^+^, the G4 structure is the most stable [57, 58].

**Fig. 1:**
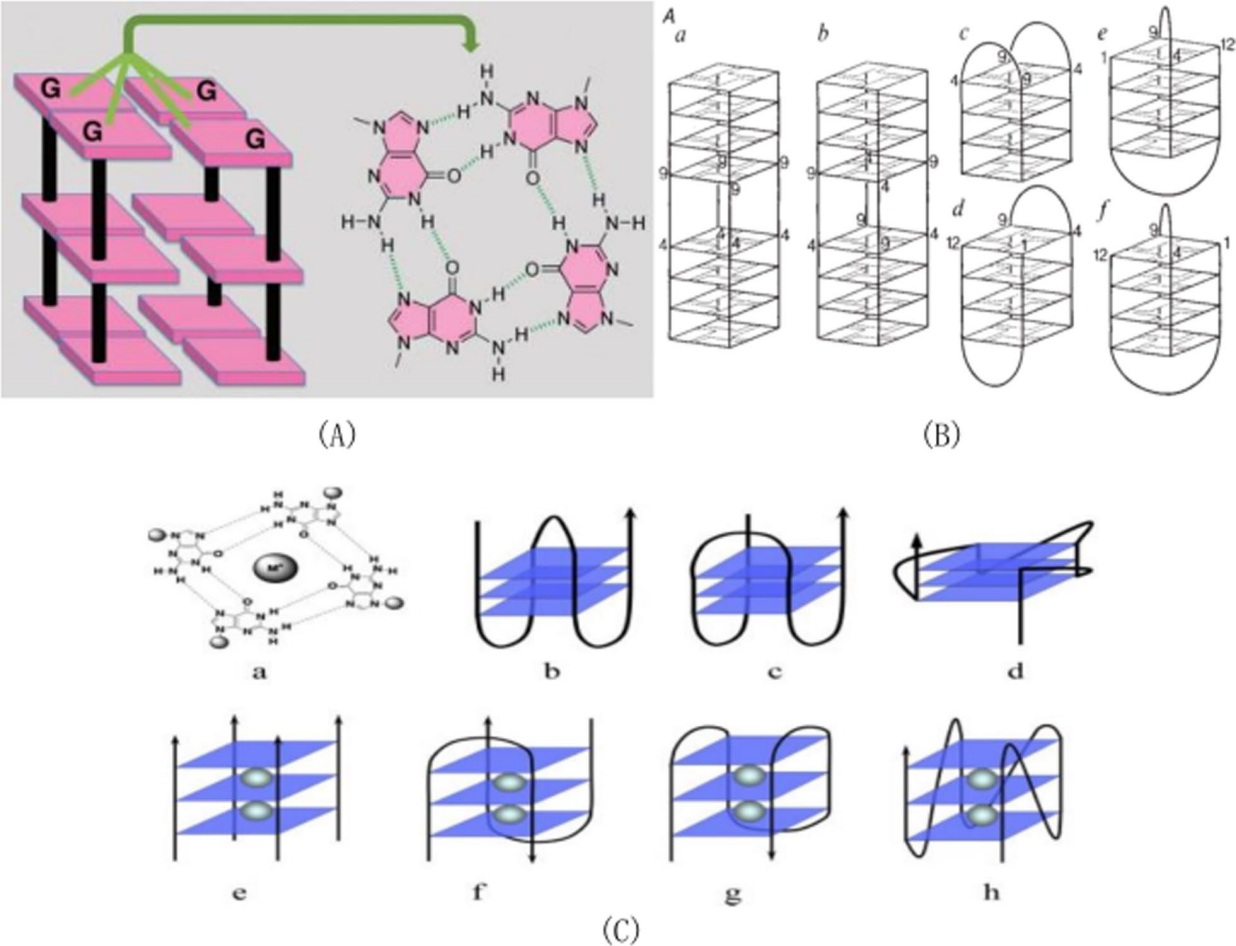
**A** G-quadruple chain plane structure. **B** DNA G-tetrastranded conformation. **C** Chair, basket and spiral conformations

The characteristics of DNA G-quadruplex chain, such as helical orientation, spatial structure, chain polarity and molecular characteristics, make it have the structural characteristics of polymorphism. According to the different directions of nucleic acid skeleton extension in the DNA G-quadruplex structure, it can be divided into parallel, anti-parallel and mixed structures. All the chains in the parallel structure are in the direction of 5′-3′, and all the chains in the anti-parallel structure are in the direction of 3′-5′. In the mixed DNA G-quadruple chain structure, there are both mutually anti-parallel chains and parallel chains. According to the difference in the number of G-base-rich single stranded DNA, DNA G-tetra-stranded can be divided into three types: monomolecular, bimolecular and four-molecule G-tetra-stranded [59]. Among them, G-quadruplex formed by two or four G-base-rich single chains is an intermolecular structure, as shown in Fig. 1A and B. Among them, bimolecular G-quadruplex is formed by the combination of two chains in the form of hairpins and polymerization, and four-molecule G-quadruplex is usually A parallel structure [59]. A single molecule G-quadruplex formed by a G-rich single chain is also called intra-molecular G-quadruplex, as shown in Fig. 1C. It can be further divided into chair, basket and spiral types according to different conformations [53]. B is Basket type, C is chair type, D is propeller type, E is four-ply parallel type, F is double-ply anti-parallel type, G is single-ply anti-parallel type, H is single-ply parallel type, in which the extension direction of the chain in chair type and basket type is anti-parallel.

### AS1411

**Fig. 2:**
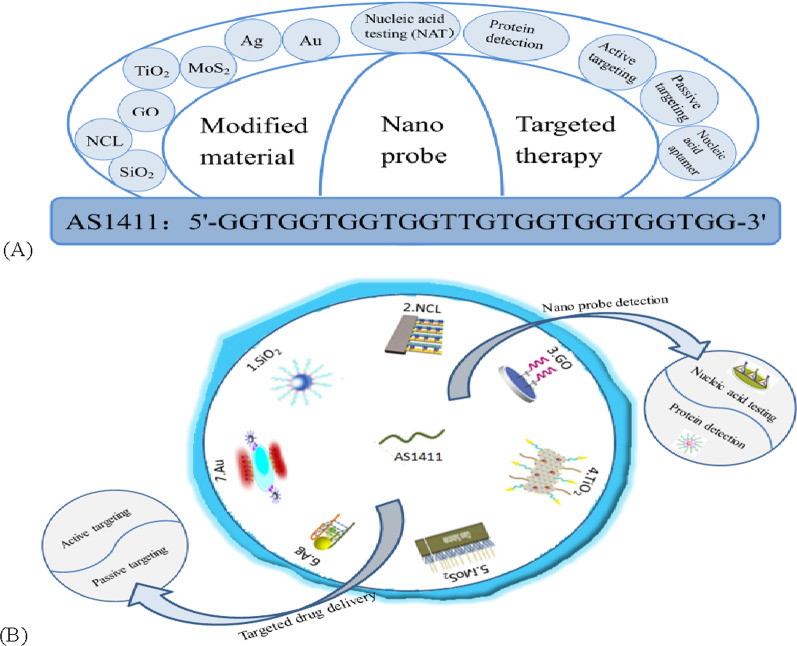
**A**The modified material of AS1411 and its general application in biomedical targeted therapy and nanoprobe. **B** Schematic diagram of AS1411 modified nanomaterials and their application in biochemistry

AS1411, formerly known as AGRO100, is a quadruplex-forming oligonucleotide aptamer application that binds to nucleoli. AS1411 (core sequence: 5′-GGTGGTGGTGGTTGTGGTGGTGGTGG-3′) is rich in the DNA sequence of G, physiological conditions can form G4 structure [46, 60]. AS1411 can cause toxicity when entering cancer cells, so it can directly kill cancer cells without adding any killing agent [42, 43, 61–64]. AS1411 may be one of the most promising nucleic acid sequences in G-quadruplex. AS1411 was incidentally found to have good nuclease resistance and thermal stability. In addition, it has shown certain anti-proliferative activity in almost all cancer cell lines, so it seems to have a wide range of therapeutic potential, and it is also the first anticancer adaptor and the first known drug specifically targeting nucleoprotein, and the phase I clinical study on it has also achieved good results [46]. Although AS1411 has a good targeted therapeutic effect, the mechanism of its cancer targeting action is not fully understood [43, 46, 65]. Other forms of DNA can exist alongside the classical double helix as viable molecular targets. In the past decade, the telomere G-quadruplet structure has been considered as a potential target for the discovery and development of novel anticancer therapies. AS1411 is a landmark G-quadruplet structure in the study of g-quadruplet structure, folding and polymorphism. Significant progress has been made in the mechanism of selective interactions between double-stranded DNA and telomere G-tetrachromes [65]. Figure 2 shows the discussion of AS1411 modified material and its application in biomedical targeted therapy and nanoprobe, which will be introduced in detail in the following paragraphs.

## AS1411 modified nanomaterials

The unique size of nanomaterials endures them with unique properties, such as small size effect, surface effect, volume effect, quantum size effect and macroscopic quantum tunneling effect, etc., and nanoparticles can have the advantage of targeting cancer by simply accumulating and intercepting in cancer cells [66–78]. The aptamer optical switch coupled with different nanomaterials has good characteristics and biosensor possibilities. The aptors can not only be used as optical switching and sensing tools, but also can be used for real-time detection and imaging. With the development of adaptor optical switches, the selectivity and sensitivity of molecular beacons have been combined with adaptor technology, which can be combined with fluorescence systems or various nanomaterials to improve their performance [79]. Due to the special chemical properties of nanomaterials, surface modification is easy, and molecules with different properties can be connected through a variety of functional groups [80].

### AS1411 binds to nucleolins

**Fig. 3:**
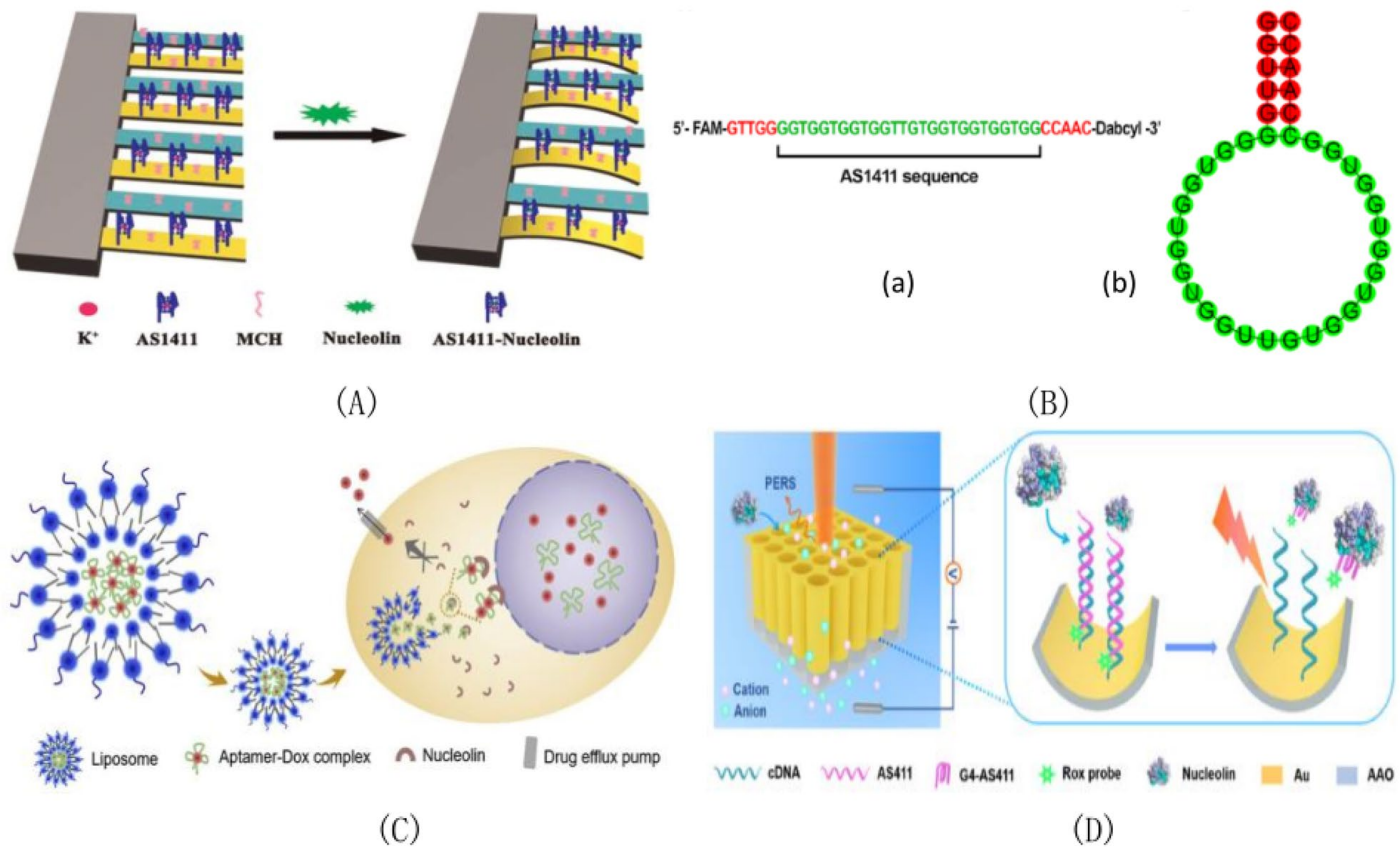
**A** Schematic diagram of A micro-cantilever biosensor for nucleoprotein detection. **B**(a) protocol for AS1411-N5 (b) protocol for expected base pairing. **C** protocol for active transport of nucleolin-mediated liposomes (Ap-Dox) to the cancer cell nucleus. **D** Schematic diagram of NCL in situ detection strategy

As a receptor protein expressed on the cell membrane, nucleoli are usually located in the nucleus and cytoplasm of phospholipins, which are widely distributed in the nucleus and cytoplasm. It has a variety of biological functions, not only participating in the synthesis of ribosomes, but also playing a regulatory role in cell proliferation, differentiation, division, chromosome replication and other processes [32, 81, 82], but its distribution morphology in each component of cells has not been obtained. Transcriptional regulation of a specific subset of miRNAs (miR-21, miR-221, miR-222, and miR-103) may be associated with the occurrence, progression, and drug resistance of breast cancer. It has been found that nucleolins are found on the surface of cell membranes, which act as anchors for binding to specific ligands. Moreover, nucleolin is highly expressed on the surface of various tumor cells (including TNBC cells) [83], but is normally expressed on the surface of some normal cells. These findings provide evidence for the idea that nucleolins can mediate the selective uptake of specific molecular receptors by tumors. It has been found that nucleolins play a unique role in the proliferation and metastasis of some tumor cells. This highlights the importance of nucleolin in cancer biology. Therefore, nucleolin becomes an ideal biomarker for the diagnosis and treatment of targeted tumors. The nucleolus can bind to specific RNA and G-rich DNA fragments through strong binding force, so that the nucleolus can also bind to the G-rich AS1411 aptamer [63, 84]. And aptamer AS1411 has been used as a transporter of small molecule drugs for targeted tumor therapy [85, 86]. Zhou et al. [34]successfully developed an current type immunosensor specifically for the identification and detection of circulating tumor cells, based on the tyramine signal amplification (TSA)-based signal enhancement system layered deposition Infinite coordinate polymer (ICPs). Kandasamy Saravanakumar et al. [87] developed a dual stimulus–response drug delivery system(DDS) for site-specific delivery of EN in the cancer microenvironment for the treatment of pH dependent aptamer AS1411 modified and erotinib loaded chitosan nanoparticles in non-small cell lung cancer. The successfully synthesized APT-EN-CSNPs complex was released by nucleolin-targeted drugs, which could improve the therapeutic efficiency of En in non-small cell lung cancer. Induction based on a variety of inflammatory and angiogenic stimuli can lead to a common vision threatening pathology of corneal neovascularization (CNV).Oscar Vivanco-Rojas et al. [88] not only evaluated the anti angiogenic effect of AS1411 on vascular endothelial growth factor-induced in vivo CNV models. We also describe the effects of AS1411 on cell migration, cell proliferation, and miR-21 and miR-221 expression of human limbal stromal cells treated with vascular endothelial growth factor (VEGF). Jing et al. [89] successfully introduced aptamer AS1411 into NCL super resolution imaging, and obtained detailed visualization of NCL distribution in nucleoli, nucleoplasm and cytoplasm at the nanometer level. Because AS1411 has better cell uptake and enhanced stability, it can realize the recognition of intracellular NCL without permeation, and display the structure of NCL in different parts of the cell in detail. This work shows the detailed organization of NCL in cells and reveals differences in the distribution of NCL between cancer and normal cells, providing a better platform for future physiological and pathological advances. The Kandasamy Saravanakumar group [90] constructed an anti-human lung cancer drug delivery system based on an aptamer functionalized polymer, which targeted drug delivery with high efficiency and did not damage normal cells. The main principle is that APT-DOX-PLGAPPP neuropeptide induces cell apoptosis by activating apoptosis related proteins, and studies the ability of APT-DOX-PLGA-PVP nanoparticles targeted drug release to improve the therapeutic efficiency through nucleolin acceptor endocytosis, which enhances the therapeutic effect of anti-lung cancer. Daniella Ishimaru et al. [91] proposed a model to illustrate the opposite effects of nucleolin and AUF1 in regulating the stability of bcl-2 gene. When nucleolin targeting aptamer AS1411 damaged the function of nucleolin in MV-4-11 cells, the binding between AUF1 and bcl-2 mRNA increased. This indicates that the degradation of bcl-2 gene induced by AS1411 is the result of interference of nucleolus protecting bcl-2 gene and recruitment of exosomes by AUF1.Rosalba Perrone group [92] tested whether aptamer AS1411 could interfere with the entry of HIV1 cells. AS1411 was tested against different virus strains, cell lines and primary cells. Li et al. [93] developed a micro-cantilever biosensor based on aptamers for ultra-sensitive marker-free detection of nucleolins. The sensor in the micro-cantilever array was suspended functionalized with AS1411 aptamer. In order to eliminate environmental interference, 6-mercapto-1-hexanol (MCH) was used to modify the reference cantilever. Because the interaction between the nucleolin and AS1411 can cause a change in surface stress, it can lead to differential deflection between the sensor and the reference cantilever. As shown in Fig. 3A, the yellow is the sensor cantilever functionalized with AS1411, and the green is the reference cantilever modified with MCH. After the injection of the nucleolus solution, the interaction between the nucleolus and AS1411 causes the surface stress to change, resulting in a different deflection of the sensor cantilever compared to the reference cantilever. This method provides an ultra-sensitive method for quantitative analysis of target proteins and detection of tumor markers. The expression of NCL aptamer AS1411-N5 in PCA cell membrane and peripheral blood mononuclear cells of patients with prostate cancer in Andre Miranda group [94]was detected by using NCL aptamer AS1411-N5. As shown in Fig. 3B(a), a fluorophore (FAM) and a quenching agent (Dabcyl) were added to each end of the AS1411 sequence, which was obtained by adding 5-nucleotide extension at the 5' end and its complementary sequence at the 3' end. Both ends were labeled at the 5'end and the 3' end with fluorophore and quenching agent, respectively, so as to obtain AS1411-N5 aptamer. As shown in Fig. 3B(b), red denotes the nucleotides that form double-stranded bodies, and green denotes the sequence AS1411. It has weak fluorescence in the absence of NCL. In the presence of NCL, the fluorescence intensity increases with the conversion of AS1411-N5 structure. Prolongs the distance between the quenching agent and the fluorophore, resulting in fluorescence emission. A nuclear targeted drug delivery system (MCF-7/ ADR) for breast cancer drug resistance avoidance based on the active transport properties of nucleolus to the nucleus and its affinity to the nucleus was developed by Li et al. [95] in 2019. The success of this system is mainly the development of a nuclear targeted liposome preparation for the loading of aptam-Dox complex. As shown in Fig. 3C, aptamer AS1411 was inserted into (Ap-Dox), resulting in DOX hydrochloride being encapsulated in the aqueous interior of the liposome (Lip (Ap-Dox)). Lip(AP-Dox) is then diffused into MCF-7/Adr cells, where the Ap-Dox complex binds to nucleolin and enters the nucleus. Among them, the role of nucleoproteins is to actively migrate to the nucleus, and the role of apperer AS1411 is to bind nucleoproteins and finally form the phenomenon of DOX enhanced nuclear uptake. Compared with other drug delivery systems, DOX hydrochloride can be effectively accumulated in the nucleus, which is used to effectively kill cancer cells and reduce the multi-drug resistance of cancer cells. A novel NCL-rich adaptive sensor based on three-dimensional hybrid plasma element material nanochannels was proposed by Shi et al. [96] in 2021 under the condition of the first application of electric field. As shown in Fig. 3D, the unique Raman characteristics of Rox dyes are used as Raman reporting agents; the cDNA labeled with 5-sulfhydryl group was fixed on the surface of the meta-material by Au–S bond, so as to realize the functional materials of binding NCL aptamers. A schematic of a 3D hybrid plasma meta-material PERS aptasensor for NCL in-situ detection strategy is shown.

### AS1411 modified graphene oxide nanomaterials

**Fig. 4:**
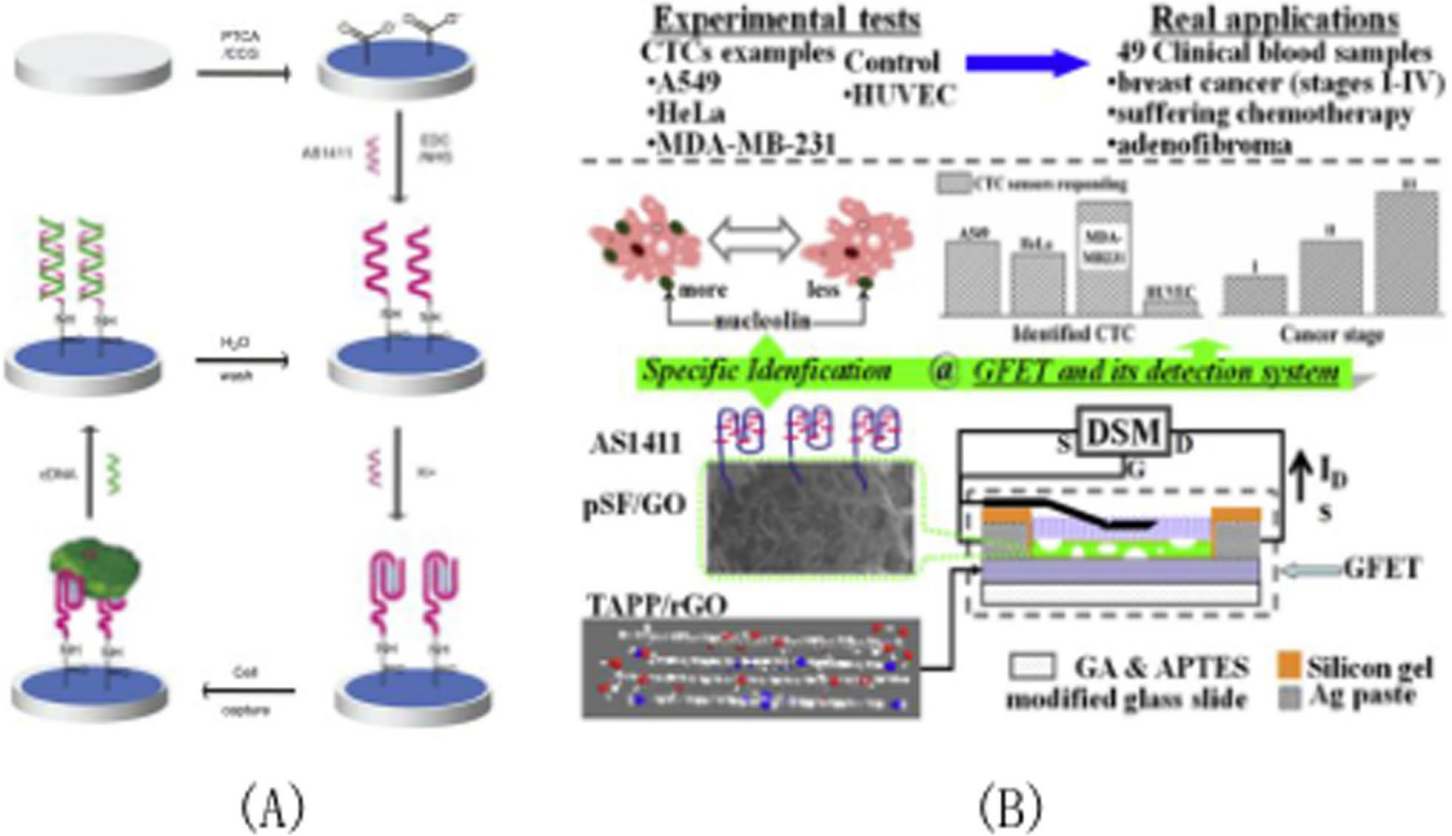
**A** Schematic diagram of A reusable aptamer/graphene-based aptamer sensor; **B** The CTCs sensor structure of TAPP/ rGO-FET was fabricated on the slides modified by GA and APTEs, and then modified by pSF/GO and functionalized by AS1411 in turn

Based on the concept of AS1411 targeting A549 cells and the concept of aptamer-modified iodide ion selective electrode sensing based on porous GO modified aptamer, Zhang et al. [97] proposed an aptamer-trapping and ion-selective electrode sensing strategy in 2018, which was inspired by ion-selective electrode cell detection technology. By combining the covalent effect of dextran with the surface of nano-GO flakes, Mona Alibolandi group [98] prepared stable dextran coated nano-Dextran coated GO in 2016. The resulting GO exhibited good biocompatibility. The GO-DEX-Apt-CUR nanocomplex was formed by loading curcumin onto deoxyredoxin mainly through π-π stowage interaction. Among them, the introduction of AS1411 aptamer is used to deoxygenate the hydroxyl group in glucose oxidase. As shown in Fig. 4A, based on A graphene-modified electrode, Feng et al. [99] in 2011 developed A novel electrochemical sensor capable of simultaneously distinguishing 1000 cancer cells from normal cells. It uses aptamer AS1411 and functionalized graphene to detect unlabeled cancer cells. The main principle of this sensor is to take advantage of AS1411's high binding affinity and specificity for nuclear proteins that are overexpressed on the surface of cancer cells. Through DNA hybridization, the e-DNA sensor can be regenerated and reused to detect cancer cells. Based on PSF/GO-TAPP/rGO-FET CTCs sensor for circulating tumor cell(CTCs)detection, Hu et al. [100] developed and integrated chemically synthesized graphene derivatives with aptamer sensing strategies in 2019. As shown in Fig. 4B, AS1411 captured by CTCs can be identified by the current response of the graphenefield effect transistor (GFET). In clinical trials, the measured GFET response was compared with the confirmed diagnosis in order to demonstrate the correctness and clinical usability of the proposed CTCs sensor.

### AS1411 modified mesoporous silica

**Fig. 5:**
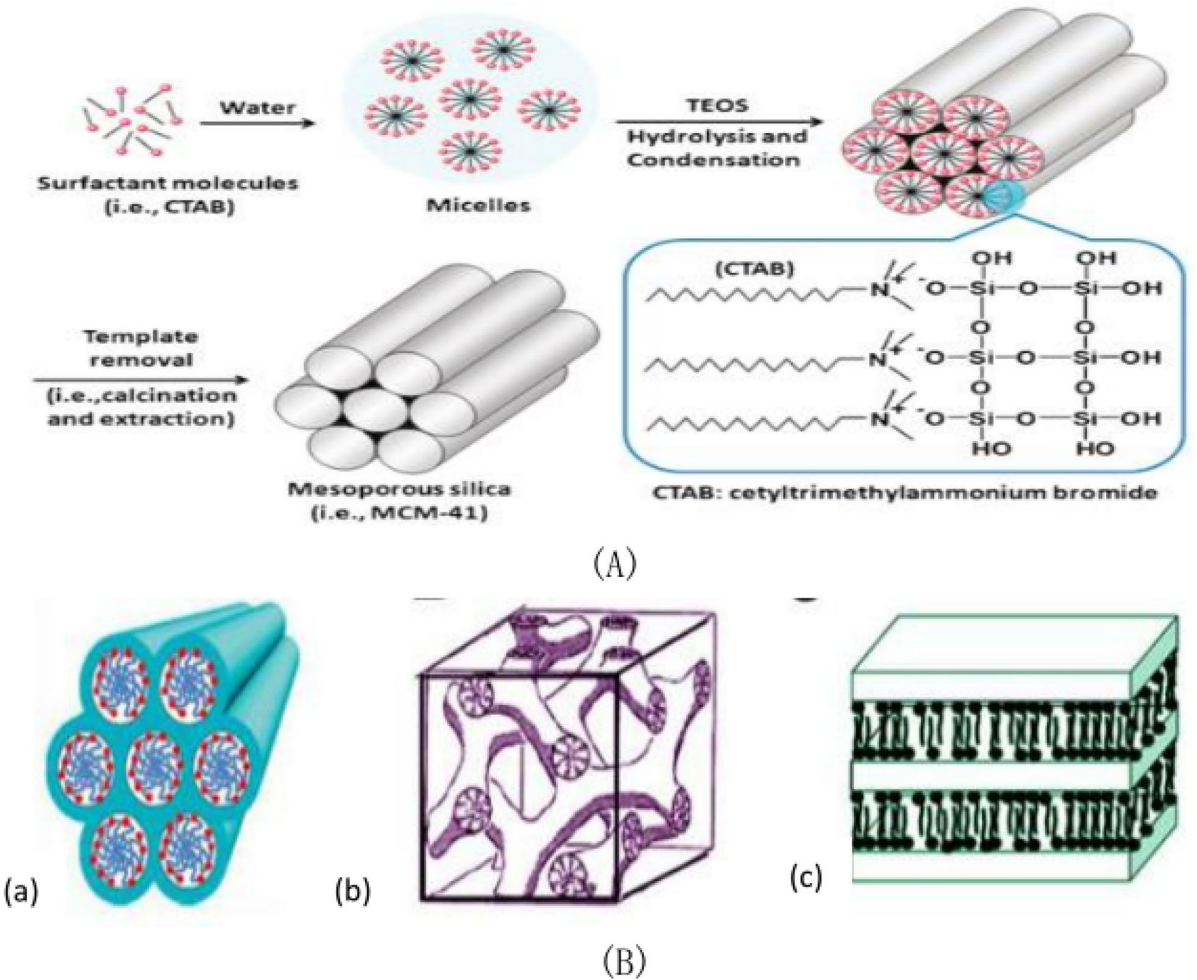
**A** Formation mechanism scheme of MCM-41 mesoporous silicon. **B** Structure arrangement of M41S: (a) MCM-41 nanoparticles: hexagonal phase, (b) MCM-48 nanoparticles: cubic phase, (c) MCM-50 nanoparticles: layered phase

The development of mesoporous silica is very rapid, and the preparation method is perfect day by day. Mesoporous silica takes surfactant as template, tetraethyl orthosilicate is hydrolyzed under the catalysis of acid or alkali to form nanoparticles, and then the template is removed by calcination or organic solvent reflux to obtain mesoporous silica nano-materials [76, 101]. The field of biomedical science requires the synthesis of MCM-41 mesoporous silicon with different properties, such as uniform shape, moderate size, appropriate pore structure and adjustable structure, as shown in Fig. 5A [102, 103]. Drug delivery system based on nano-mesoporous silica as carrier can ensure zero drug leakage before reaching the lesion site, but can achieve targeted drug release at the lesion site, which is conducive to improve the efficacy and reduce the toxic and side effects of drugs, and has a broad application prospect in clinical treatment and medical care. It has become a research hotspot in biochemistry, nanoscience, medicine and materials science. The field of biomedical science requires the synthesis of MCM-41 mesoporous silicon with different properties such as uniform shape, moderate size, appropriate pore structure and adjustable structure [104–106]. Figure 5B shows the structural arrangement of M41S.

**Fig. 6:**
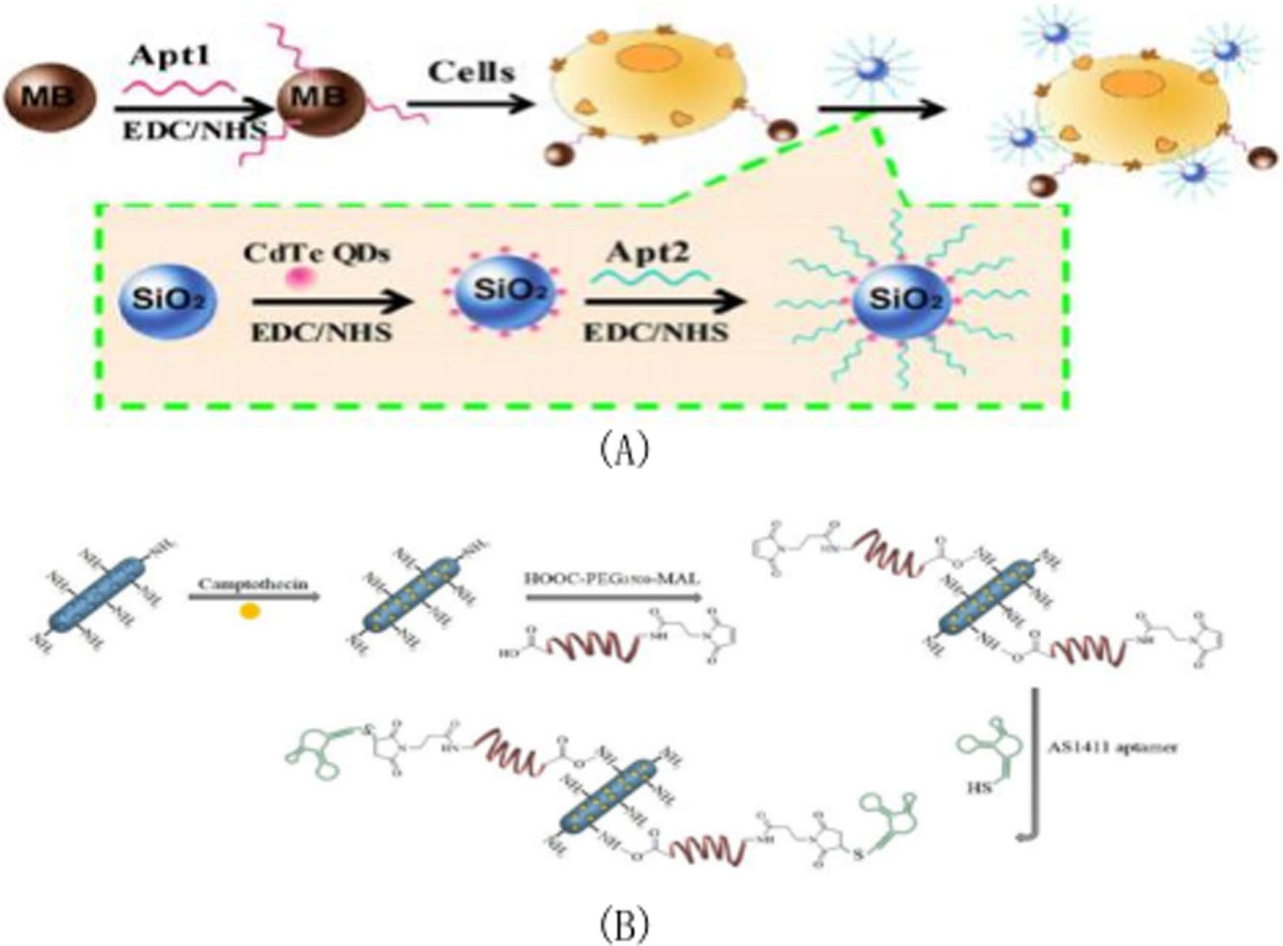
**A** MCF-7 cells were isolated and detected using magnetic beads functionalized with MUC1 aptamers and AS1411-modified quantum dots loaded on the surface of SiO_2_ nanoparticles. **B** Schematic diagram of the synthesis method of aptamer binding drug delivery system

The Fahimeh Charbgoo group [107] used a divalent aptamer composed of ATP and aptamer AS1411 to target mesoporous silica nanoparticles with a trapezoidal structure with bi-functional molecules. This approach not only provides the targeted ability to deliver DOX assurance to the tumor, but also has supporting gating properties in delivering the drug to the cell. Based on selective collection of aptamer-cell interactions, Chua et al. [62] developed a new method for detection of breast cancer cells (MCF-7) in 2013. As shown in Fig. 6A, this method mainly captures and labels MCF-7 cells through interactions between aptamers and cell surface receptors. Compared with other methods, this method achieves selectivity by using aptamer of mucin 1(MUC1) (APT1) and aptamer AS1411(APT2) as recognition elements. Because the silica nanoparticles are wrapped by quantum dots, the signal is amplified to a certain extent, thus improving the detection sensitivity. The polyethylene glycolated rod-like mesoporous silica nanoparticles were developed by Maryam Babaei team [108] in 2020 as co-delivery vectors for both thectothecin and Survivin shRNA expressing plasmids. Figure 6B is a schematic diagram of the synthesis method of aptamer binding drug delivery system. The mesoporous silica nanorods were synthesized by the Stober method at room temperature using a simple green chemical modification. To guide delivery to colorectal cancer, the AS1411 aptamer marker preparation system was used. The hydrolysis and condensation reactions are carried out in ammonia water with the silica precursor as tetraethylorthosilicate (TEOS) and the template as cetyltrimethylammonium bromide (CTAB). Mesoporous silica nanoparticles(MSNs)transformation is achieved by hydrogen bonding and non-polar interactions between TEOS and cetyltrimethylammonium bromide templates. The reason why it can be surface modified with different molecules is that the silanization of hydroxylated MSNRs with (3-aminopropyl) trie-thoxysilane(APTES) as the linker forms a siloxane network resulting in the amino-terminal group protruded from the surface. Surfactant concentration, temperature, ionic strength and pH of medium reaction are particularly important because of the need to control the shape and length of nanoparticles to be rod-like.

### AS1411 modified silver nanoparticles

**Fig. 7:**
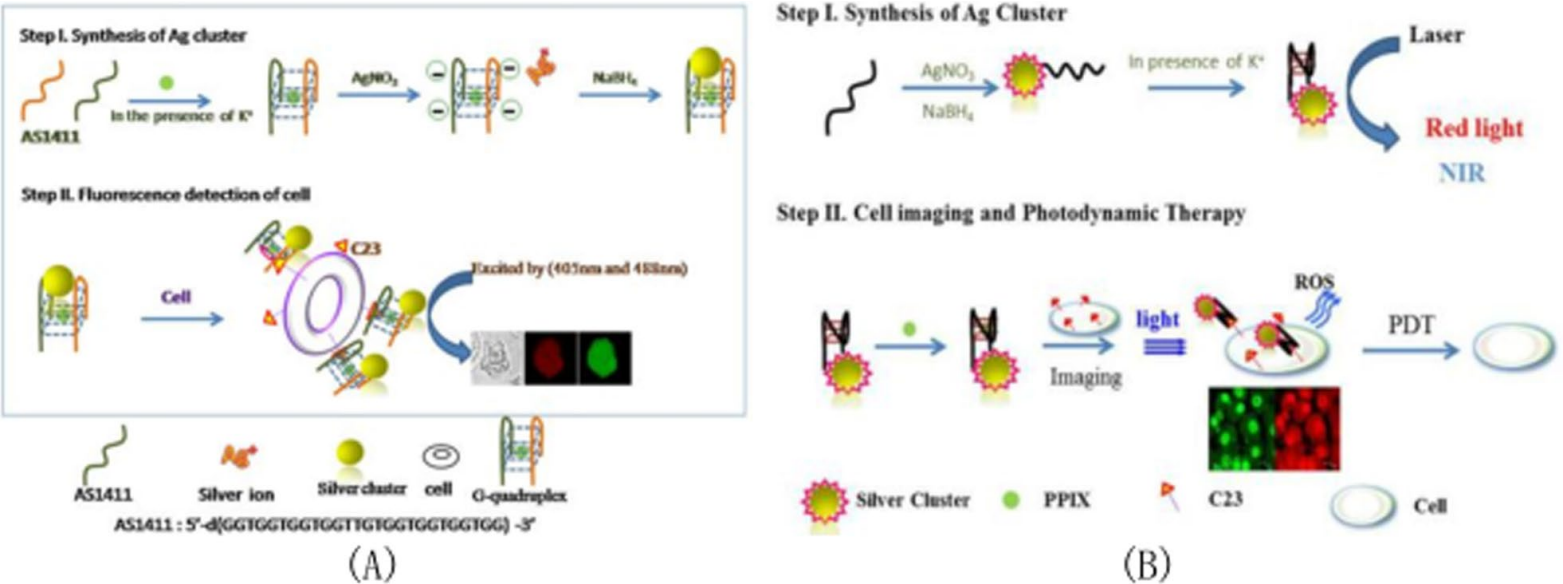
**A** Schematic diagram of fluorescence biological imaging based on silver nanoclusters. **B** Principle demonstration of multifunctional sodium chloride for HeLa cell bio-imaging and photodynamic therapy

With the development of nanotechnology and synthesis methods, the preparation methods of silver nanomaterials have been developed to a certain extent. At present, the main synthesis methods include chemical reduction method, electrochemical method, hydrothermal method, polyol method, etc. [109]. Chemical reduction method is the most studied synthesis method, which has been successfully used in the preparation of silver nanomaterials with different morphologies. Chemical reduction method is simple in operation and has a high yield. Generally, metal salts are used as precursors, and appropriate reducing agents are used to reduce metal salts into target nanoparticles under controlled conditions. One-pot method was used by Li et al. [110] in 2016 to synthesize aptam-labeled polychromatic silver nanoclusters, namely aptam-labeled AS1411 nanoparticles and aptam-labeled MUC1 nanoparticles, which emitted green light. The green and yellow emission characteristics of silver nanoparticles are mainly determined by the surface oxidation state of silver nanoparticles. Specific cell imaging mainly depends on the ability of AS1411 aptamer and MUC1 aptamer to bind to cancer cells. By simply mixing the G-quadruplex template with silver ions and the reducing agent NaBH_4_, Ai team developed a method that can directly form fluorescent silver nanoclusters for biological imaging of HeLa cells [111]. As shown in Fig. 7A, the fluorescence biological imaging steps of silver nanoclusters are shown. Step 1: Guanine-rich oligonucleotides form G-tetraplex in the presence of potassium ions. Therefore, silver nanoclusters are generated by the reduction of silver ions and quadruplex by NaBH_4_. Step 2: Bioimaging of cells incubated with silver nanoclusters using confocal spectroscopy. Ai et al. [112] prepared water-soluble DNA-coated silver nanoclusters with near-infrared fluorescence that can be used for both targeted cancer imaging and enhanced photothermal therapy or photodynamic therapy. The main reason for the effect of such nanoclusters is the synergistic effect between photodynamic reagents and near-infrared silver nanoclusters. As shown in Fig. 7B, NIR-AgNCs-AS1411 synthesized from the DNA chain of the AS1411 sequence as a template is mainly used for biological imaging, while multifunctional nano-conjugates(MF-NACs)are produced by combining NIR-AgNCs-AS1411 with photosensitizer PDT-PPIX. In the presence of K^+^, PPIX can effectively capture near-infrared AgNCs-AS1411 and apply it to photodynamic therapy (PDT).

### AS1411 modified gold nanoparticles

Gold nanomaterials have a good application prospect in a variety of biomedical applications, in which the synthesis of different shapes of gold nanomaterials can be applied to cancer diagnosis and therapeutic agents [76, 113, 114].

#### Passive targeting

By adjusting the sequence and concentration of the DNA template, Zhang et al. [115] 2020 proposed a programmable near-infrared window plasma modulating gold nanoradiation that could successfully achieve local surface plasmonic resonance (LSPR) for red-shift gold radiotherapy. The gold nano-dumbbells prepared in the gold radiotherapy have good thermal stability, strong photo acoustic signal and high photo thermal conversion efficiency. Targeted-gold nano-radiotherapy (Ap-AuND) was obtained by binding gold nano-dumbbells to nucleolin-targeted aptamer AS1411. This targeted Ap-AuND can effectively image subcutaneous 4T1 tumors and significantly inhibit tumor growth and stimulate effective host anti-tumor immunity. Gold nanomaterials were synthesized from doxorubicin-rich gas chromatographic inserts (hairpin DNA) and AS1411 modified gold nanoparticles. In 2020, Zhang's team [116] developed an active targeted drug delivery for the treatment of colon cancer and the chemical photothermal effect triggered by near-infrared laser. Among them, DOX release of hairpin structure is realized by using photothermal effect (PTT) effect. The interaction between AS1411-nucleolin is the main reason for the active targeting of AAHD-nanoparticles to SW480 cells. Studies have shown that the gold nanosys can not only significantly inhibit the growth and proliferation of colon cancer cells, but also have good targeting and synergistic anti-tumor ability. Zahra Khademi et al. [117] proposed a targeted drug delivery system that combined DOX and aptamer targeting Fokkhead box M1(FOXM1 Apt) for joint delivery to cancer cells, in which the carrier was CS-AUNPs and the target ligand was AS1411 aptamer. DOX and FOXM1 Apt were then delivered to A549 and 4T1 cancer cells. Experimental results show that the combined product of FOXM1 Apt and DOX can not only significantly improve the efficacy, but also have low toxicity and side effects. And the resulting complex can selectively carry its therapeutic agent into cancer cells, thereby reducing the systemic effects of DOX. In 2019, Samaneh Kabirian-Dehkordi group [118] used optimized AS1411-conjugated gold nanoparticles (GNS-AS1411) to inhibit nucleolin expression at both RNA and protein levels. Based on the affinity between AS1411 cancer cells and the over-expressed nuclear protein receptor in cancer cells, Yasaman-Sadatborghei team [119] developed a simple but highly sensitive colorimetric method for detection of cancer cells in 2016. The specific binding of AS1411 to the target cells then results in the removal of the aptamer from the solution. There are two conditions: when there is no aptamer in the solution and the complementary ssDNA probe is hybridized, the visible red solution is produced. The absence of target cells or the presence of normal cells results in the coexistence of ssDNA probes and aptamers in the solution, resulting in the formation of DNA-aptamers, producing a purple solution visible to the naked eye. The Marzieh Baneshi group [120] showed that AS1411 aptamer functionalized albumin nanoparticles developed on iron oxide and gold nanoparticles could significantly inhibit the proliferation of cancer cells without showing any toxicity. Compared with other non-targeted nanoparticles, functionalized AS1411 aptamer nanoparticles can improve cell uptake and efficiency in MCF7 breast cancer cells due to the aptamer's high affinity for the overexpressed nuclear protein on the surface of MCF7 cells. A near-infrared (NIR) light-absorbing hollow gold nanosphere for targeted photothermal therapy of tumors in combination with AS1411 aptamer was developed in 2018 [121]. AS1411-polyethylene glycol-AuNC apers were synthesized by combining AuNC functionalized by polyethylene glycol and AS1411. Selective uptake of breast cancer cells is possible due to the selective binding of AS1411 to nucleoli. In other words, the results show that the aptam-bound antigen is an effective tumor targeting photothermal agent. In 2018 [122] the clinical potential of NCL/miR-221/NFκB/DNMT1 in targeted acute myeloid leukemia nanotherapy was demonstrated by targeting gold nanoparticles with nuclear localization signaling (NLS) peptides.

**Fig. 8:**
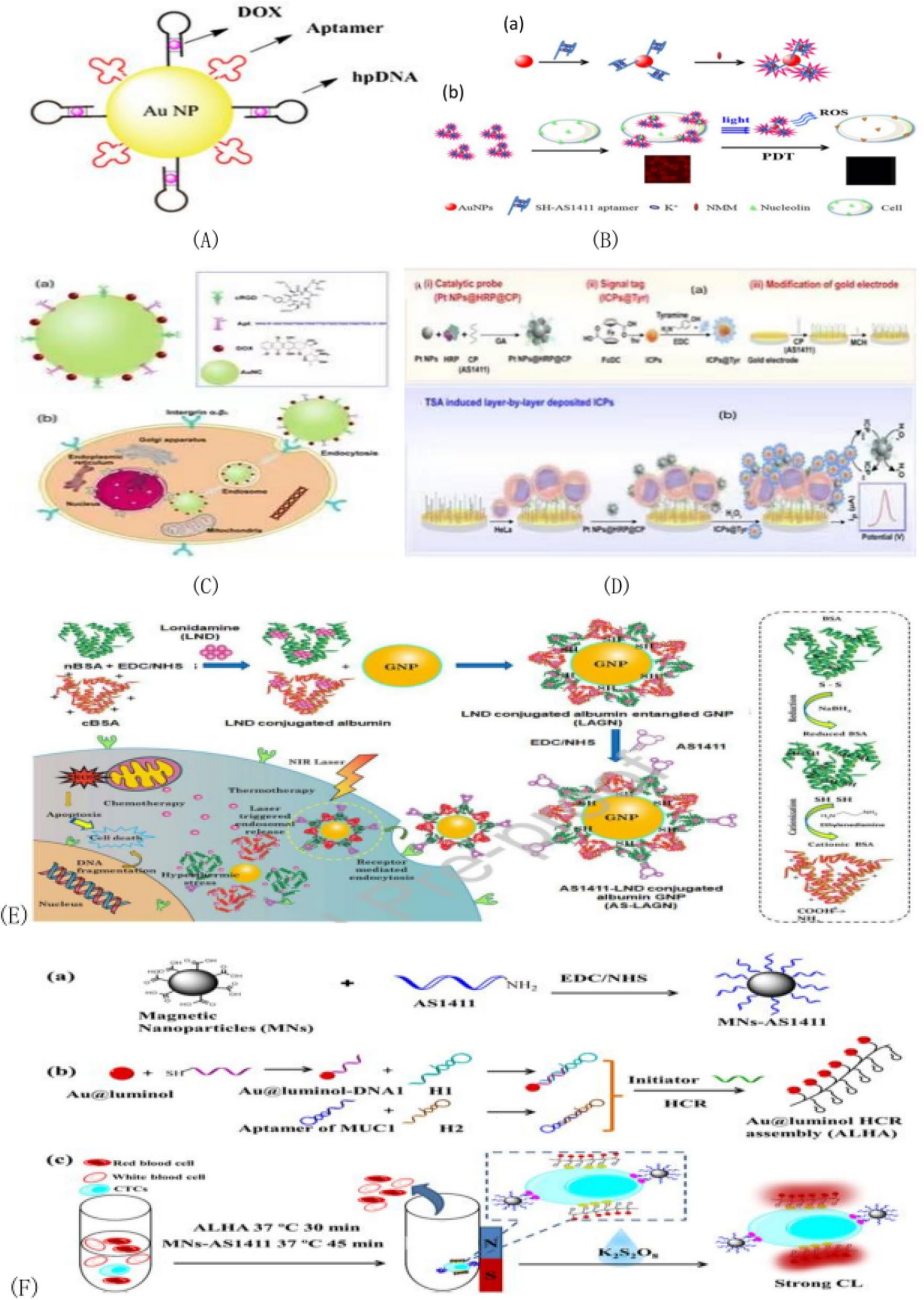
**A** DOX-APT/hp-AuNP nanocomplexes; **B** (a) Synthesis of NAANPs by binding functionalized AS1411 aptamers to NMM. (b) Detection of cancer cells with overexpression of nucleolin on cell surface using targeted cell imaging and PDT; **C** Nanostructure schematic diagram of (a) Aunc-dox-CrGd-APT (b) endocytosis process schematic diagram of AuNC-dox-CrGd-APT; **D** CTCs were detected by electrochemical immunosensor. **E** Schematic diagram of aptamer and clonidamine-bound albumin modified gold nanoparticles (As-LAGN). **F** Schematic diagram of the experimental process

An aptamer/hairpin DNA-gold NP(APT/HP-AuNP) conjugate was designed to deliver drugs to the surface of antigens by oligosulfide units [114]. As shown in Fig. 8A, the aptamer's role is targeted delivery, and the anticancer drug DOX is loaded into the repeated sequence of hp-DNA. AuNP-based nanocomposites provide an ideal platform for light-controlled drug delivery in cancer therapy. As shown in Fig. 8B(b), Ai et al. reported the development of a multifunctional AS1411 fluorescent gold nanoparticles (NAANPs) that were successfully applied to targeted cancer cell imaging and highly effective photodynamic therapy. Figure 8B(a) shows that the citric acid templated gold nanoparticles were mixed with SH-AS1411 aptamer to form the Au–S bond, thus forming the mercaptoacetic acid nucleic acid functionalized gold nanoparticles. In the presence of K^+^, G-quadruplex is formed and binds to N-methylmesoporphyrin IX (NMM). Figure 8B(a) shows fluorescence imaging of the test NAANPs targeting cancer cell HeLa in the presence of a specific immune response between AS1411 and an overexpressed nuclear protein on the cancer cell surface. Among them, gold nanoparticles act as carriers and enhancers in the experimental process. They can not only maintain the inherent cytotoxicity of cancer cells, but also promote the absorption of fluorescent groups in cells. In this way, targeted cell imaging has clear efficiency [123]. Chen et al. [124] developed and reported a novel double-targeted nanoplatform (AuNC-cRGDApt) for tumor diagnosis and treatment. As shown in Fig. 8C(a), nanometer conjugates with histidine as AuNC@His stabilizer were synthesized. Histidine plays a key role in the binding of nanoclusters and the functionalization of small molecules due to its carboxyl and amino functions. As shown in Fig. 8C(b), nanocomposites can be internalized into the cytoplasm through receptor-mediated endocytosis, and then effectively delivered to the nucleus to enhance the anti-tumor ability. Because cyclic RGD (cRGD) and Apt, two of the functional ligands, have a high affinity for the integrin αvβ_3_ and nuclear protein in tumor cells. Based on nano-assembled gold nanoparticles (GNP) with ketamine (LND) and aptamer AS1411(AS-LAGN) multifunctional nanoplatform, Hma Bindu Ruttala's team [125] has developed a new, simple, biodegradable multifunctional nanodetection system for cancer therapy in 2020. As shown in Fig. 8D, in this sensor system, horseradish peroxidase and nucleolin- targeted aptamer bifunctional platinum nanoparticles have improved catalytic performance and catalyze tyramine-labeled electroactivity signals to report deposition on tumor cells. In the presence of HeLa cells, platinum nanoparticles @horseradish peroxidase catalyzed H_2_O_2_ will reduce to produce a large amount of O_2_, resulting in a significant increase in the peak current of ICPs reduction. ICPs deposited layer by layer on the electrode sensing interface based on the generated current and TSA can be used to quantify the number of cells and improve the sensitivity of HeLa cell detection. As shown in Fig. 8E, AS1411 aptamer was bound to the surface of the nanoparticles through its specific affinity for the nucleoprotein receptor, and this binding method significantly improved particle accumulation in cancer cells. The constructed multifunctional protein coronal gold nanoparticles (GNP) combined with clonidamine (LND) and aptamer AS1411(AS-LAGN) nanoparticle assembly not only showed significant tumor regression properties, but also showed enhanced anti-tumor efficacy, which could be used for phototherapy and chemotherapy in cancer treatment. Cao et al. [126] established a simple, convenient and economical enzymy-free chemiluminescence cycling method for detecting CTCs in whole blood for tumor cells in 2020. In this assay, Fe_3_O_4_ magnetic nanospheres (MNs) were first modified with AS1411 nucleoside aptamer to capture and isolate CTC in blood samples. CTCs were identified and isolated from whole blood samples using magnetic nanoparticles modified with CTCs specific aptamer AS1411. The Au@luminol nanoparticle HCR assembly (ALHA), which contains multiple MUC1 aptamers and a large number of CL tags, acts as response signal and specific recognition element for sensing CTC and detects CL signal. As shown in Fig. 8F(a), Fe_3_O_4_ magnetic nanospheres (MNs) were modified with AS1411 nucleoprotein aptamer in order to capture and isolate CTCs from blood samples; In order to identify CTCs(MCF-7 cells) and generate detectable chemiluminescence signals, numerous multi-molecular aptamers and a large number of Au @ Luminol nanoparticles chemiluminescence tags were included in the Au @ Luminol HCR module (ALHA) as shown in Fig. 8F(b). As shown in Fig. 8F(c), based on the good chemiluminescence performance of Au@Luminol-K_2_S_2_O_8_ system, this method has the characteristics of high sensitivity, simple operation and low cost.

### AS1411 modified molybdenum disulfide (MoS_2_)

**Fig. 9:**
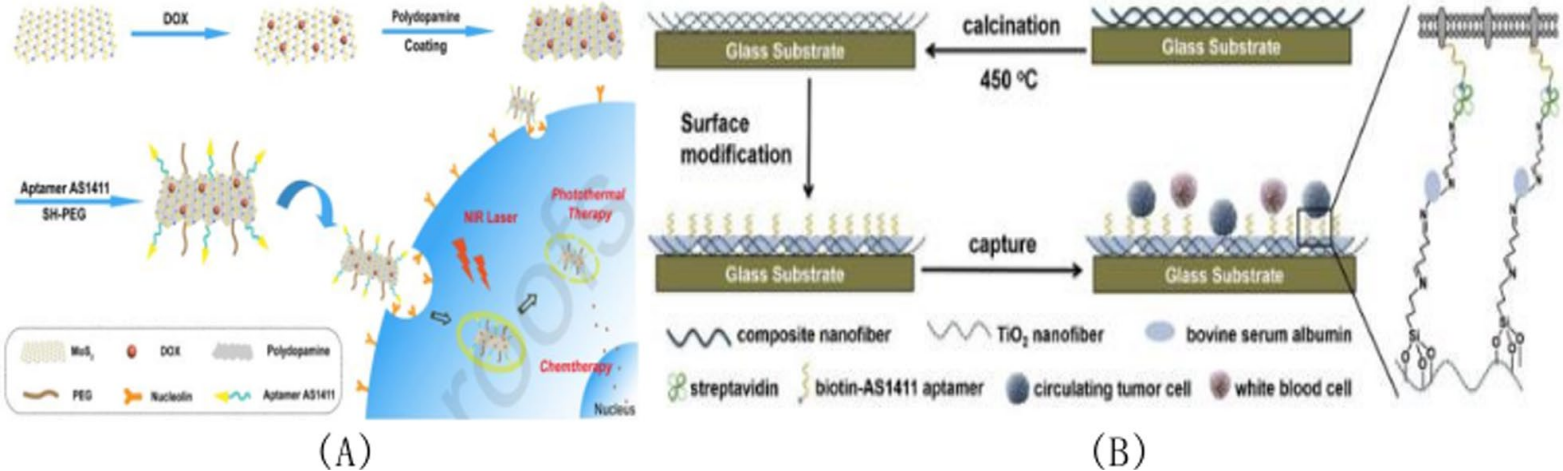
**A** Schematic diagram of aptamer modified molybdenum disulfide nanosheets. **B** Schematic diagram of the capture of circulating tumor cells at the biological interface of TiO_2_ nanofibers

Based on MoS_2_ and apter-functionalized DOX-APT-PEG-PDA-MoS_2_ nano-drug delivery platform, a novel tumor cell targeted lysosomal acid environment/ near-infrared laser dual-response drug delivery system for targeted synergistic chemotherapeutic photothermal therapy of breast cancer was developed in 2020 [127]. Molybdenum disulfide nanomaterials containing doxorubicin (DOX) and coated with polydopamine (PDA) layer are used as the carrier of DOX and photothermal agent for photothermal therapy (PTT). Figure 9A shows A schematic diagram of aptamer-modified MoS_2_ nanosheets. As shown in the Fig. 9, the prepared DOX@Apt-PEG-PDA-molybdenum disulfide nanomaterial can combine with and enter breast cancer MCF-7 cells through the mediation of aptamer AS1411. Molybdenum disulfide and PDA can convert the 808 nm near-infrared laser into heat energy, playing the role of PTT. The nanoplatformer loaded with DOX can then be released by tumor lysosomes in an acidic environment and stimulated by 808 nm near-infrared laser irradiation. The DOX@Apt-PEG-PDA-MoS_2_ drug delivery system developed provides an excellent platform for targeting and synergistic chemo-thermal therapy of breast cancer cells.

### AS1411 modified titanium dioxide (TiO_2_)

Titanium, as a non-toxic transition metal, can be used to construct a variety of nanomaterials. Due to its excellent characteristics of structure, composition and physical and chemical properties, titanium-based nanomaterials have broad potential for biomedical application [128]. Based on a novel CTCs detection strategy of electrospun nanofibers and nucleoprotein aptamer AS1411, Liu et al. [129] developed a cheap TiO_2_ nanofiber matrix for efficient, sensitive, and specific capture of CTCs for efficient and selective capture of circulating tumor cells from heterogeneous samples. The titanium dioxide nanofiber substrate was prepared by electro-spinning combined with calcination. As shown in Fig. 9B, bovine serum albumin (BSA) was used as the blocking molecule and nucleoprotein aptamer AS1411 was used as the cell trapping agent. The nanofiber substrate surface was modified with anti-adhesion molecule bovine serum albumin (BSA) and aptamer AS1411, the role of which was to specifically bind the nuclear protein overexpressed on the surface of cancer cell membrane. Based on TiO_2_ nanofiber substrate, it can demonstrate high efficiency and specificity in capturing nucleolin-positive cells through synergistic action. Therefore, with the joint assistance of bov-serum albumin and aptamer AS1411, the functional interface of TiO_2_ nanofiber prepared can efficiently and specifically capture CTCs in samples.

## Application of AS1411 modified nanomaterials

**Fig. 10:**
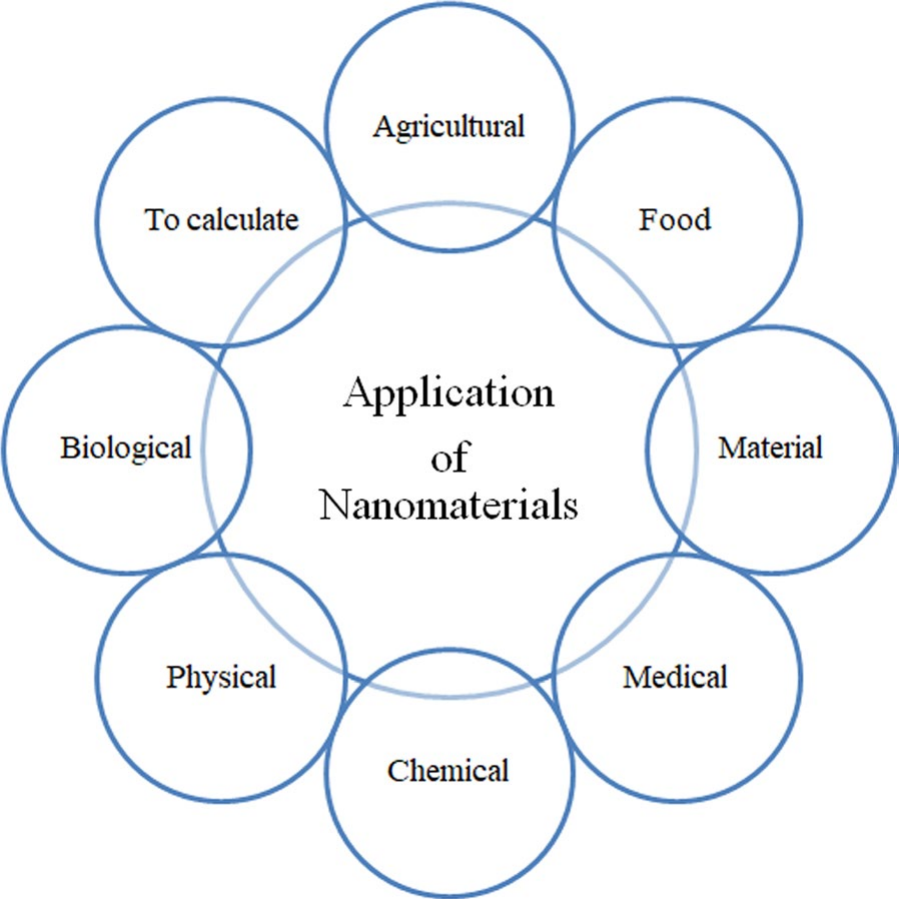
Multidisciplinary applications of nanotechnology

Nanomedicine is the science of applying nanotechnology to the prevention, diagnosis and treatment of diseases. The development of nanotechnology in various aspects is shown in Fig. 10. The development of nanotechnology has provided a variety of nanomaterials and nanomedicines, which can be applied in various fields and greatly affect our lives [130]. Among them, the unique advantages of nanomaterials provide new ideas for the targeted treatment of cancer [30, 131, 132].

### Biomedical applications of AS1411 modified nanomaterials

AS1411 is a well-known guanosine-rich oligonucleotide used mainly in clinical trials for the treatment of leukemia. This guanosin-rich oligonucleotide has a variety of biological properties, such as increased cellular uptake, enhanced nuclease resistance, antiviral and anti-proliferative effects. It has a stable three-dimensional structure and can bind to nucleoproteins through shape specific recognition, the anticancer aptamer AS1411 is a guanosin-rich oligonucleotide used in clinical trials for the treatment of leukemia. Oligonucleotides are constantly searched for target proteins for the purpose of studying these screening [46, 133], and AS1411 can also lead to overstimulation of megakaryocytes in cancer cells [134]. AS1411 aptamer can also be used for targeted delivery of therapeutic agents and imaging agents for cancer cells [43]. AS1411 can bind to plasma membrane nucleoprotein highly expressed in different cancer cell lines to induce tumor cell death, and can be used in the treatment of acute myeloid leukemia (AML) and renal cell carcinoma (RCC) [135, 136]. In DU145 prostate cancer cells, AS1411 induced the activation of EGFR, Akt, p38 and Rac1 [81]. AS1411 was used as an identification element, and HeLa cells were selected as model cells for early and accurate diagnosis and treatment of cervical cancer, ITO/TiO_2_/ZnIn_2_S_4_ electrode was formed as PEC substrate, and the aptamer AS1411 and amphoterionic peptide were fixed by the connection of polydopamine (PDA). The PEC cell sensor was used to detect HeLa cells. Among them, zwitterionic peptide can improve the antifouling ability of PEC cell sensor in real biological media, and 6-mercaptohexanol (MCH) is used as blocking molecule. The photocurrent changes caused by the apparent steric hindrance of the target cells were detected quantitatively [137]. AS1411 can be linked to strep tomavidin through the biotin bridge to target nucleoli. After in vivo injection, fluorescently labeled AS1411 is targeted to photoreceptors and retinal pigment epithelium and other retinal cell types [138]. AS1411 is a nucleoprotein targeting aptamer that has been tested as an anticancer agent in Phase II clinical trials [139].Various nanomaterials have been used to deliver different kinds of drugs and probes in the treatment of diseases of the axial nervous system [140]. In order to achieve a better application prospect in 786-0 molecular magnetic resonance imaging of renal cancer, the AS1411-polyethylene glycol-manganese dioxide nanoprobe formed by covalent coupling reaction was prepared in 2018 [141] using the stability of polyethylene glycol-manganese dioxide nanoparticles. The target molecule AS1411 modified by amino group was coupled with PEG-MnO nanoparticles to prepare the AS1411-PEG-MnO nanoprobe, which could clearly display 786-0 renal cancer cells on magnetic resonance imaging in vitro. The newly generated probe has a longer retention time in vivo in 786-0 renal cancer tumors than the PEG-MnO nanoprobe. Most importantly, the nanoprobe can be removed from the body not only through intravenous administration, but also through the renal clearance pathway. Zhang et al. [142] used luciferin labeled aptamer AS1411 to modify unlabeled fluorescent silicon nanopotts by cross-linking agent covalently. The ratio pH biosensor was constructed, in which the unlabeled silicon nanocrytes acted as a carrier and fluorescence reference. Based on the biosensor's small size, low toxicity, excellent pH reversibility and good photostability, this multi-functional biosensor is beneficial for applications in intracellular environment and biomedical analysis. Based on the AS1411-functionalized poly (L-gamma-glutamine)-paclitaxel(PGS-PTX)nanoconjugate, In the 2017, Luo [143] constructed an effective anti-glioma drug delivery system (AS1411-PGS-PTX) to precisely target paclitaxel binding and optimize solubility. Mona Alibolandi et al. [144] used polyethylene glycol-glycolic acid with different functions as the linker between polyamine-amine G5 dendrimers and nucleolin-resistant AS1411 ssDNA aptamers. A polyethylene glycolated PAMAM dendritic molecule for selectively delivering CPT to nucleolin-positive colorectal cancer cells (HT29 and C26) was synthesized. In order to target site-specific colorectal cancer cells with overexpressed nucleoprotein receptors, this dendritic molecule was functionalized with AS1411 anti-nucleoprotein aptamer with the goal of inhibiting C26 tumor growth in vivo and significantly reducing systemic toxicity. Jessica Lopes-Nunes group [145] introduced two novel targeted delivery methods based on two different conjugated systems: the first was a conjugated G4-structured aptamer AS1411 with near-infrared probe indo-cyanine green (ICG), and the second was a conjugated complex of AS1411-ICG with acridine orange ligand C8. In vitro and in vivo experiments in a mouse melanoma model indicate that this conjugated system can be used to study rapid fluorescence and biological distribution in vivo in real time. AS1411-ICG and the complex of AS1411-ICG conjugated with acridine orange ligand C8 showed significant toxicity in cultured melanoma B16 cells and could be effectively absorbed by melanoma in mice. Therefore, this AS1411 conjugate can be used as a drug targeting agent for melanoma cancer models. Guo and others based on synthesized dipeptide [146] self-assemble ability of new multi-drug drug nanoparticles (DNPs/Clolar/AS1411/HA/RNA/DOX). Three kinds of anticancer drugs, nucleoside anticancer drug Clolar, small interfering RNA and chemotherapy drug DOX, were selected to form multifunctional nanoparticles with DNPs drug carrier by chemical bonding, Watson Crick base pairing and π–π stack interaction. The main reason for the synthesis of DNPs/Clolar/AS1411/ HA is that the carboxyl group on DNPs is coupled with the hydroxyl group and amine group of Clolar or HA. Among them, the aptamer AS1411 was used in combination with DNP to improve the targeting ability of nanomaterials. Hyaluronic acid is a polypeptide chain that induces membrane fusion. In neutral environment, no membrane fusion activity was observed. In an acidic environment, the structure of hyaluronic acid changes dramatically, resulting in interaction with the endosome, resulting in exposure of more affinity ends of the membrane, which fuses with the membrane, leading to the escape of substances in the endosome. Synthetic small molecule siRNA is a double-stranded RNA molecule that induces gene silencing, and its main role is to guide complementary messengers to degrade or silence RNA after transcription.

**Fig. 11:**
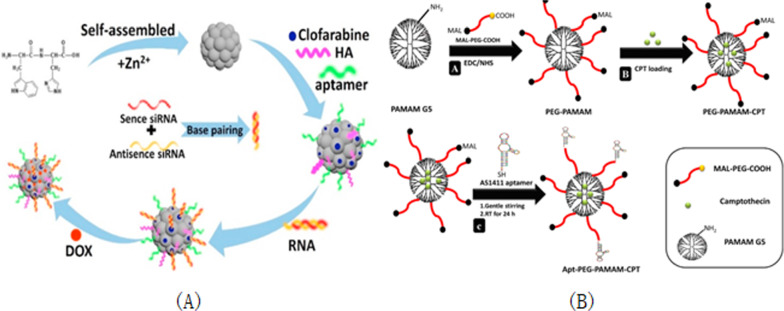
**A** DNPs Clolar/AS1411/ HA/RNA/DOX design. **B** Synthetic diagram of polyethylene glycolated polyamide-amine dendrimers

As shown in Fig. 11A, using by thymidine and molecular recognition between thymidine analogs connect siRNA to cells, the chemotherapy drug doxorubicin DOX accumulated through the π–π interaction and aromatic nitro phenol accumulation, the formation of DNPs/Clolar/AS1411/HA/RNA/DOX, fluorescence intensity decrease at this time. When the nanomaterial reaches the cytoplasm, a biochemical reaction takes place in the cell to decompress the carrier, and the anti-tumor drugs attached to the carrier are released and put into effect, while the fluorescence intensity resumes. This nanomaterial can be used for in vivo and in vitro imaging. Based on the ability of AS1411 aptamer/hyaluronic acid bifunctional microlatex to co-contain paclitaxel and docetaxel (AS1411/SKN&DTX-M), Wang [147] penetrate the blood–brain barrier and inhibit the growth of in-situ gliomas by targeting CD44/nuclide overexpression in gliomas in 2019. Figure 11B shows A schematic synthesis of A polyethylene glycolated polyamide-amine dendrimer. By a two-step reaction of EDC/NHS Chemistry, polyamide-amine with a molar ratio of 1:4: polyethylene glycol binds hetero-functionalized polyethylene glycol (MAL-PEG-COOH) to the surface of the polyamide-amine dendrimer. In the first stage, the carboxyl group of mercapto polyethylene glycol is activated and converted to sulfamate. In the next stage, it was conjugated with the amino sulfonic acid surface group of the polyamide-amine dendrimers and successfully polyethylene glycolated to prepare polyacrylamide gel [144].

### Targeting study of modified nanomaterials with AS1411

As AS1411 contains 26 bases and is rich in guanine (G), it can form an antiparallel G-quadruplex structure that specifically binds to its target protein nucleolin (also known as C23) [148–150]. C23 is a phosphorylated protein with the highest content in the nucleoli of eukaryotic cells, which is related to nucleolar genesis, cell growth and reproduction, and ribosome synthesis. In normal cells, C23 is mainly present in the nucleus. In tumor cells, C23 is overexpressed in the cell membrane as well as in the nucleus. AS1411 not only has a certain spatial structure (about 2.4 nm), which can block the diffusion of drugs, but also can specifically identify nucleolus proteins that are overexpressed on the surface of cancer cells [151], increasing the whip-direction of the nano-carrier. If the anticancer drug delivery system is equipped with AS1411 target head, the active targeting of the anticancer drug delivery vector can be greatly increased [152, 153].

### Advantages of targeted research on AS1411

The combination of drug carrier and free small molecule anticancer drugs constitutes a drug delivery system, and the physical and chemical properties of drug carrier itself can significantly change the original mode of action of anticancer drugs, such as its distribution and metabolic behavior in vivo [9, 16, 17, 35, 48, 63, 66–68, 70, 82, 150, 154–176].

The ideal drug carrier should have the following characteristics: (1) a long enough circulation time in the human body (stealthy in the blood) to avoid being detected and cleared by the immune system, which requires biocompatible surfaces, such as PEG grafted surfaces [177]; (2) Carrier design has been developed from the initial passive targeting design [178, 179] to the active targeting construction of particles at the present stage (modification of wheel groups on the surface of particles, such as mono antibody, folic acid, aptamer, etc.) [180–182]. (3) The drug carrier only releases the drug at the lesion site. This may involve the stimulation-responsive design of drug carriers, i.e., releasing drugs to targets under the instructions of physical signals (temperature, magnetic field, light, etc.) and chemical signals (pH, ionic strength, chemicals, etc.) [183–185]. The modifiable AS1411(Ap) generates a DNA apperer of a specifically highly expressed nucleoprotein in blood vessels, which can form an anti-glioma delivery system with paclitaxel-loaded PEG-PLGA micelles [186]. Nucleolin apperer AS1411 can be used to in situ functionalize the carrier to improve the delivery and uptake of the complex to target cells [187]. Drug delivery using AS1411 generally has several advantages over other targeting strategies [188], including: (1) because the aptamer size is much smaller than that of monoclonal antibodies, a higher molecular density of aptamers can be attached to each particle; (2) Because AS1411 is chemosynthetic rather than biological, particles attached to AS1411 are easier to manufacture and store than antibodies; (3) AS1411 can target a variety of cancers, while many targeting agents are specific to a single tumor class; (4) AS1411 does not have immunogenicity, so it will not produce immune rejection in human body.

### Targeting application of AS1411

**Fig. 12:**
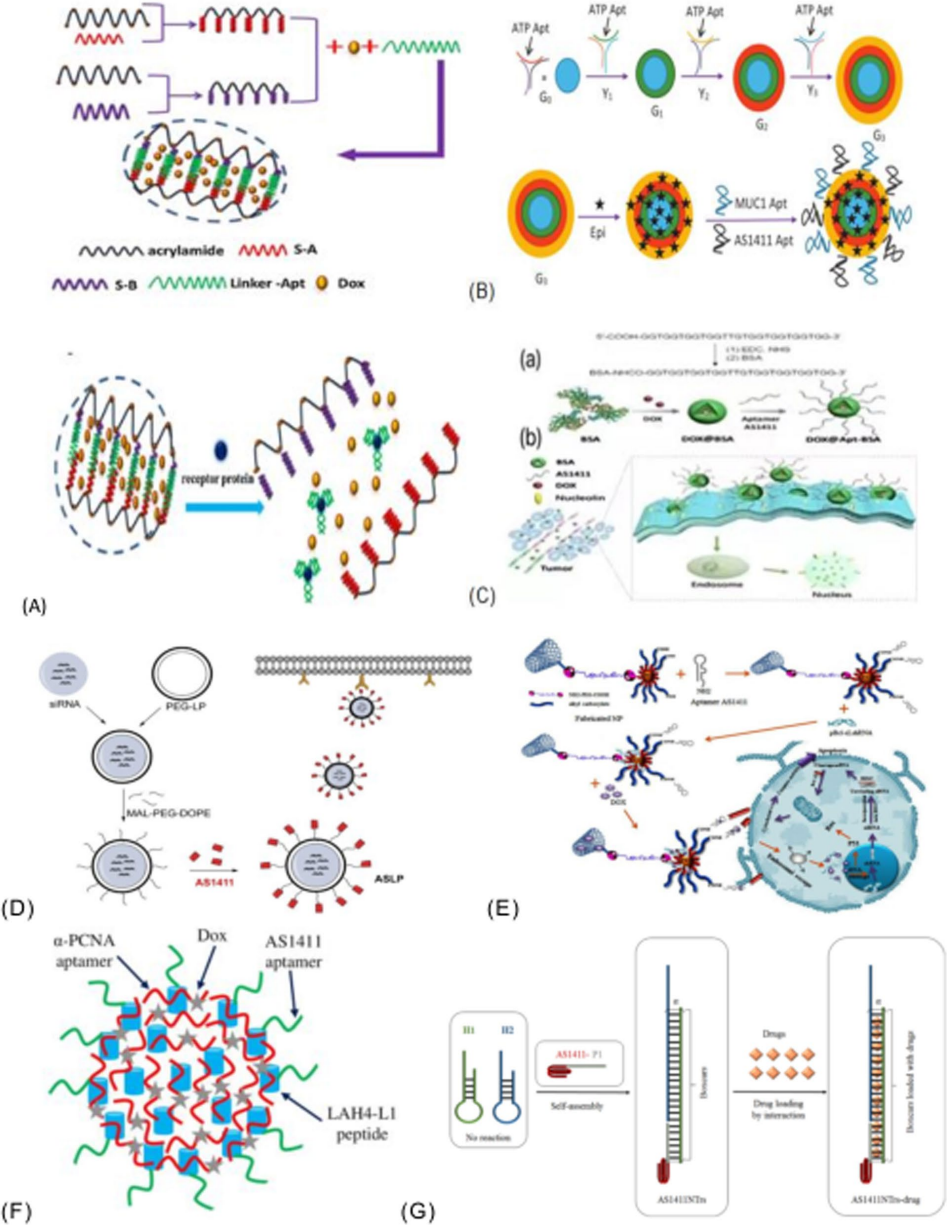
**A** Synthesis process of aptamer functionalized hydrogel. **B** The working principle of targeted drug delivery of aptamer functionalized hydrogels. **B** Schematic diagram of APTs—dendritic macromolecule (G3)-EPI complex formation. **C **DOX@Apt-BSA Preparation diagram of nanoparticles and drug delivery system. **D** Schematic diagram of siRNA delivery using AS1411-conjugated (ASLPs). **E** Schematic diagram of co-delivery of Bcl-XL shRNA and DOX to gastric cancer cells using AS1411-bound NP. **F** Schematic diagram of AS1411 modified LDPNs. **G** Self-assembly of AS1411NTRS transporter molecules

Jia et al. [189] constructed a novel surface-functionalized drug nanocarrier for tumor targeting and enhanced drug delivery based on bimetallic fluorescence NiCO-PBA@Tb^3+^ nanomaterials. poly(ethyleneglycol)-dimethacrylate (PEGMA) was functionalized by silicon atom transfer free radical polymerization (ATRP)and AS1411 was introduced to specifically identify breast cancer cells. The drug-carrying nanocarrier NiCo-PBA@Tb^3+^@PEGMA @AS1411 can show good biocompatibility and pH response, and the addition of AS1411 can enable rapid drug release in cancer cells, enhancing the tumor targeted delivery (DOX) of doxorubicin. It has been shown that the complex NiCo-PBA@Tb^3+^@PEGMA@AS1411 @DOX can accumulate in large amounts in tumor tissues. So that it can effectively inhibit tumor growth. The Yoon Young Kang group [190] designed a single and double aptamer linear DNA structure with AS1411 aptamer and MUC-1 (MUC-1) in order to achieve effective intracellular uptake and to examine the appropriate polymorphism and type of aptamer in linear nucleic acid structure. The polyvalent linear DNA structure contains dual aptamers, forming the polyvalent aptamer-fixed DNA-RNA hybridization structure. This hybridization structure can not only be specifically used as the carrier of therapeutic nucleic acids, but also show significant superior cell internalization without causing obvious systemic toxicity. Farnaz Sadat Mirzazadeh Tekie group reported the preparation of the resistance with nucleoli [191] suitable body AS1411 modified TD-miR(redox-responsive miR-145 conjugated thiolated dextran) and chitosan polyelectrolyte complexes, used to enhance the cancer cells to absorb nanoparticles, including targeted agent for attached AS1411 suitable body nucleolus resistance element. This method has higher gene silencing and apoptosis than previous methods. An approach for glioma therapy that completely overcomes both the blood–brain barrier (BBB) and the glioma barrier was developed in 2014 [192]. This is a dual-targeted drug delivery system that contains AS1411 aptamers (for glioma targeting) and TGN peptides (for BBB targeting) modified nanoparticles (ASTNPs). This dual targeted drug delivery system can improve the survival rate of mice with glioma in experiments. Based on the rheological characteristics of hydrogels, they can be used to control encapsulation and release of cancer drugs. The developed hydrogel can be regulated by sol–gel conversion in the presence of AS1411 active modifier. In 2015 [193], an active modifier functional hydrogel was prepared. The main principle of hydrogel formation is that AS1411 initiates the hybridization of acid-modified oligonucleotides, leading to gel dissolution in the presence of the target protein nucleoprotein. Such aptam-functionalized hydrogels can be used for targeted cancer cell recognition and targeted drug delivery. As shown in Fig. 12A(a), two DNA fragments, acrylate modified oligonucleotide A(S-A) and acrylate modified oligonucleotide B(S-B), are copolymerized with acrylamide to form the linear DNA-polyacrylamide conjugates PS-A and PS-B, respectively. When mixed in equal amount, AS1411 complementary to S-A and S-B sequences was added to initiate hybridization between S-A and S-B and aptamer sequences. With the progress of hybridization between S-A and S-B and the aptamer sequences, the cross linking ratio of polyacrylamide increases and the viscosity of the polymer solution increases. As shown in Fig. 12A(b), AS1411 binds to the introduced target protein nucleolin, and the cross-linking density is reduced due to the competitive target-aptamer binding, resulting in the gel dissolution and release of doxycycline wrapped in the hydrogel. Seyed Mohammad Taghdisi group [194] reported a targeted delivery system for epirubicin (Epi). As shown in Fig. 12B, the system used three aptamers (MUC1, AS1411 and ATP aptamers) to jointly design a modified and promoted dendriform macromolecule. The resulting Apts-dendritic macromolecule-Epi complex can effectively inhibit tumor growth in vivo. Xu group [195] constructed a functionalized albumin-based nanoparticle for effective drug delivery and targeted cancer therapy. As shown in Fig. 12C, the hydrophobic drug doxorubicin (DOX) was used to induce bovine serum albumin (BSA) through hydrophobic interaction, leading to the formation of stable nanoparticles by self-assembly, and then tumor targeting aptamer AS1411 was incorporated into the DOX-loaded BSA surface. Due to the specific recognition between the tumor-targeted aptamer AS1411 and the overexpressed receptor on tumor cells, higher cell uptake capacity and stronger cell efficacy against MCF-7 cancer cells were demonstrated. DOX@bovine serum albumin nanoparticles were modified by amidation with targeted ligand aptamers to form DOX@Apt-bovine serum albumin nanoparticles. A cationic liposome has been developed [196] for targeting overexpressed nuclides on the surface of A375 cells by bifunctional polyethylene glycol binding covalently to AS1411. As shown in Fig. 12D, the preparation scheme of ASLP and the targeting effect of AS1411 on tumor cells are shown. This new method of delivering tumor-targeted liposome insertion is used to treat melanoma with anti-BRAF siRNA (siBraf). By comparison, the accumulation of siRNA in tumor cells was significantly higher than that in other cells, which confirmed that AS141 1ePEG-liposome(ASLP) can show excellent tumor targeting ability. Among them, ASLP/mitogen-activating factor inhibits melanoma growth in A375 tumor xenograft mice. Based on carbon nanotube nanoparticles, the Sahar Taghavi group [33] developed an aptamer-modified co-drug delivery system for targeting tumors. The synergistic effect of Bcl-xL shRNA with doxorubicin (DOX) was studied, and AGS and L929 cell lines were selected to evaluate the efficacy of the treatment in vitro. The developed system not only has precise targeting, but also the ability to bind to nucleolin receptors that are overexpressed on the surface of AGS cells and to the nanoneedle structure of single-walled carbon nanotubes. As shown in Fig. 12E, cells were incubated into cells with free DOX or nanoplatform-containing Bcl-xL shRNA and DOX. DOX and Bcl-xL shRNA have good anticancer effect. In 2020, a new nanotherapeutic platform for co-delivery of drugs to tumor cells was designed.

The Seyed Mohammad Taghdisi group [197] is primarily using α-PCNA aptamers as therapeutic aptamers, with doxorubicin (DOX) as a subcellular pathway for chemotherapy. The α-PCNA aptamer loaded with deoxyribonucleic acid was condensed into a short cationic amphiphilic peptide using LAH4-L1. As shown in Fig. 12F, AS1411 aptamer was coated on the surface of LDPNs(Dox-loadedα-PCNA was condensed by LAH4-L1 as a short cationic amphipathic peptide) as a targeting agent in order to realize the treatment delivery system of tumor synergistic therapy. Experiments have shown that AS1411-modified low density lipoprotein can not only remain stable in the serum, but also accumulate effectively in the tumor site. The resulting complex of doxorubicin and α-PCNA(AS1411 and LAH4-L1) can be used as a novel chemotherapy method for breast cancer with few side effects and great clinical value. Pei et al. [198] prepared and characterized AS1411-tethered DNA nanostrands by a hybrid chain reaction (HCR). Moreover, the interaction of DOX, EPI or DAU with arsenic was studied by calorimetry and spectroscopic method, and was verified by agarose gel electrophoresis. As shown in Fig. 12G, the self-assembly process of AS1411 NTrs (AS1411 aptamer-tethered DNA nanotrains) transporter molecule drug is illustrated. The aptamer self-assembly process includes aptamer trigger chain (P1) and hairpin chain (H1 and H2). Among them, the role of nucleic acid aptamer is to target the nuclear protein of cancer cells, and the spontaneous polymerization and cross-hybridization of H1 and H2 is initiated by P1.

### Nanodelivery system classification of aptamer-targeted tumors

At present, reducing pain, improving the accuracy of drug therapy and targeting anti-cancer drug delivery carriers are the focus of research. It is mainly divided into the following three categories, and all of them target tumor tissue sites through the targeting effect of aptamers on tumor markers to carry out relevant diagnosis or treatment of tumors [11, 12, 33, 112, 199–214].

Nanodelivery of aptam-targeted tumors is generally divided into three categories, the specific classification is shown in Table 1:

**Table 1:** Classification of nano-delivery systems for aptamer targeting tumors

Nano delivery system	Principle	Targeted content	Refs	
Passive targeting	The aptamer is modified onto the surface of the drug-carrying nanocarrier to form the aptamer targeting nanocarrier	Targeting tumor markers by aptamers to target tumor tissue sites can be used to diagnose or treat tumors	[2, 214–216]	[9, 16, 17, 22, 37, 62, 92, 95, 98, 107–109, 111, 124, 125, 168–170, 209–225]]
Active targeting	The aptamer is directly linked to the drug/fluorescent substance to form an aptamer-drug/fluorescent substance conjugate		[2, 16, 38, 118, 173, 217, 219]	
Nucleic acid aptamer	The aptamer itself acts as a drug carrier to form an aptamer-drug assembly		[6, 72, 202–207, 207–209, 221–223]	

#### Passive targeting

**Fig. 13:**
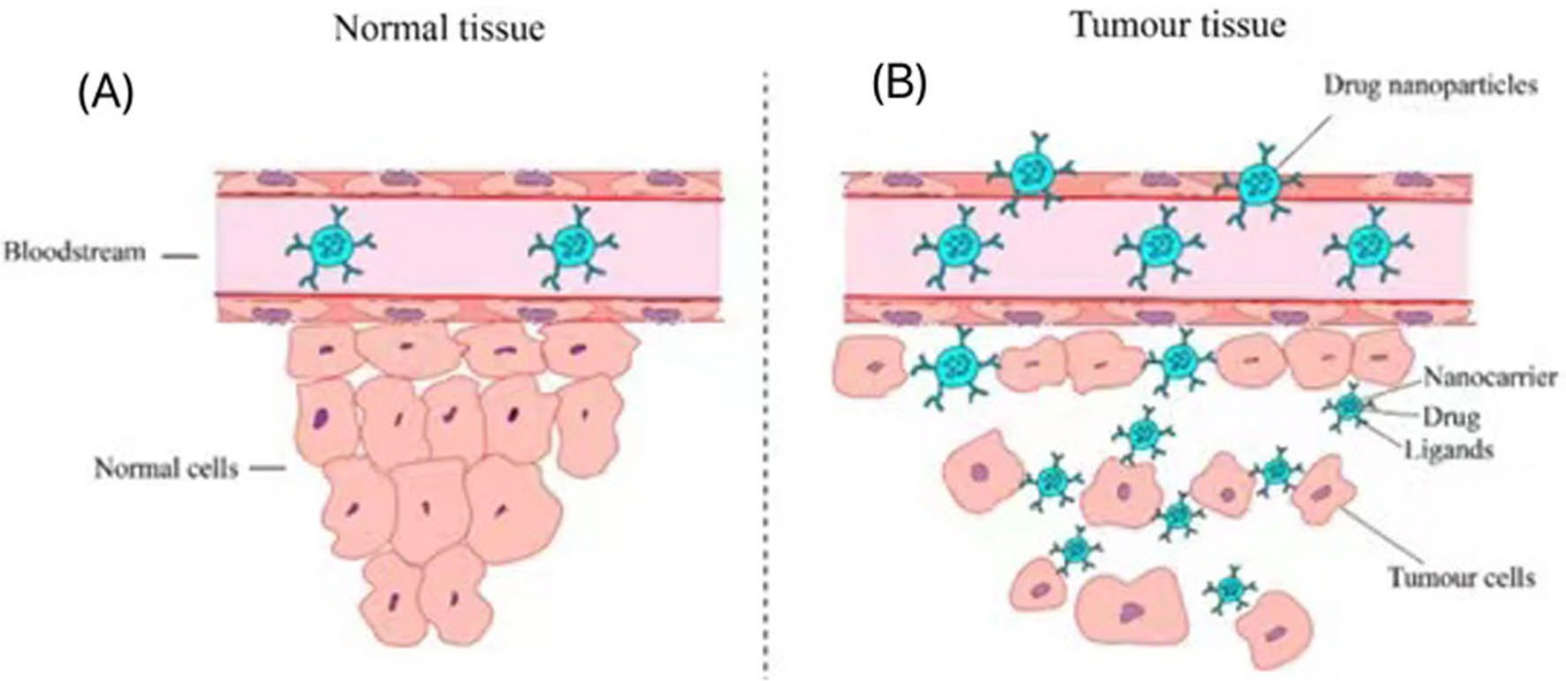
llustrate active (**A**) and passive (**B**) tumor targeting Mechanisms [2]

Passive targeting is mainly to modify the aptamer to the surface of the nanocarrier which can carry drugs to form the aptamer targeting nanocarrier. The growth and metabolism of tumor tissue is extremely vigorous, and vascular remodeling is incomplete. There is a gap between vascular endothelium of 10–1000 nm, so only particles within this range can reach the tumor site through blood circulation, through EPR effect, namely the high permeability and retention effect of solid tumor. Accumulate in tumor site is therefore whether can realize the EPR effect researchers have designed passive targeting carrier directly measure, the particle size of nano carrier starts by the kidneys to filter out, too easy to be liver intake rather than in the tumor position, therefore the particle size of nano carrier must be within the scope of a specific clearance can penetrate vascular endothelial cells, through passive targeting to enter tumor tissue, The structure and status of micro vessels in tumor sites also have a great influence on EPR effect [215, 216]. A novel aptamer-modified immunolipoprotein nanostructure for drug delivery and scale-up chemotherapy immunotherapy was proposed in 2018 [217]. The lipophilic aptam-immune adjuvant CpG fusion sequence (Apt-CpG-DSPE) was combined to facilitate the modification of high-density lipoprotein (HDL), and then doxorubinin (Dox) was sequentially embedded into the consecutive base pairs of Apt-CpG. imHDL/Apt-CpG-Dox was synthesized. This immune HDL nanotaph causes a degree of site-specific structural collapse in the material between the tumor cells that inspire HDL biological function. At this point, under the recognition condition of AS1411 and nucleolus, the dissociated Apt-CpG-Dox would be endocytosis into tumor cells and transfer Dox to the nucleus, and the released CpG motifs would further trigger antigen recognition, resulting in tumor ablation and antigen release as much as possible, while inducing the secretion of a large number of pro-inflammatory cytokines. So as to enhance the host's anti-tumor immunity. Figure 13A shows [2] how passive targeting works. In normal tissue, drug- laden nanoparticles would flow through the bloodstream, while the drug-laden nanoparticles would seep into the tumor due to the vasculature through which they leak.

#### Active targeting

Active targeting is mainly to directly connect the aptamer with the drug/fluorescent substance to form the conjugate of aptamer-drug/fluorescent substance. Pass drug delivery system is mainly based on the receptor and the ligand specificity combined into tumor cells, most species of tumor cells contain high compared to normal cells express or express receptors, so the corresponding ligand modification on the surface of nanometer carrier, can increase the tumor cells on the specificity of the vehicle recognition, greater intake of carrier, The targeted drug release can be carried out more accurately at the tumor site, with minimal or no toxicity to non-tumor tissues. In addition to improving drug availability, tumor treatment can be more effective. At present, active targeting drug delivery system is the most common to target tumor cells [16, 77, 218–221]. Mona Alibolandi group [84] developed a molecular drug delivery system for the targeted treatment of non-small cell lung cancer (NSCLC). This system uses gemecitabine (GEM) loaded AS1411 apper surface modified polyethylene glycolic acid (lactic acid co-glycolic acid) as nano polymer (Apt-GEM-NP). The working mechanism of active targeting [2] is shown in Fig. 13B. It is assisted by ligands that recognize tumor cells on the surface of the nanoparticles and bind to them specifically.

#### Nucleic acid aptamer

Nucleic acid aptamers have good stability and can be used to protect precious metal nanoclusters. Precious metal nanoclusters refer to several to dozens of Au, Ag, Pt and so on. A cluster of metal atoms. Its particle size is generally less than 2 nm [13]. An oligonucleotide sequence capable of binding to a target with high specificity. It can act on metal ions, small molecule compounds, proteins, and cells. Due to its high selectivity to target objects, AptRNA can endue the delivery system with targeted specificity, and meanwhile increase the enrichment of delivered drugs and imaging reagents in tumor tissues, which has great application potential in biomedical fields such as molecular diagnosis and in vivo targeted therapy [6, 64, 203, 204, 208, 222–227]. DNA nanostructures are considered as an ideal tool for cancer diagnosis and treatment due to their controllable appearance, biocompatibility and multiple modification capabilities [228]. Nucleic acid aptamers can improve the targeting ability of nanoparticles, mainly because aptamers have unique advantages [229]: Compared with antibodies, aptamers have the advantages of being easy to be synthesized in large quantities in vitro, good reproducibility, small molecular weight, high stability and easy to store, no immunogenicity, easy modification and reusability, etc., which have been widely used in various fields and shown good effects [229, 230]. Similarly, AS1411 aptamer also has excellent properties such as strong affinity, high specificity, cheap preparation, high efficiency and speed, high stability, small size, easy modification, low immunogenicity and good permeability. To prepare DOX-resistant breast cancer cell lines (MCF-7/ADR) targeting overexpressed nucleoprotein receptors. Liposomes containing doxorubicin (DOX) and ammonium bicarbonate (a foaming agent) were functionalized in the liposomes of the anti-nuclide aptamer AS1411 in the Liao group [231]. Among them, the main reason for the functionalization of AS1411 is to increase the affinity between liposomes and nucleoprotein receptors, promote specific binding, and thus make it less difficult for liposomes to enter tumor cells.

### AS1411 generates functionalized nanoprobes

Fluorescent probe generates fluorescence signal under specific excitation light, so as to perform qualitative or quantitative imaging and analysis on the measured object [232]. Fluorescence imaging and analysis methods have high sensitivity, good specificity, small sample size, convenient and rapid operation, and have unique advantages in the field of trace biochemical analysis and detection. They are widely used in food detection, environmental safety detection, human disease related diagnosis and analysis and other fields.

#### Nucleic acid testing (NAT)

**Fig. 14:**
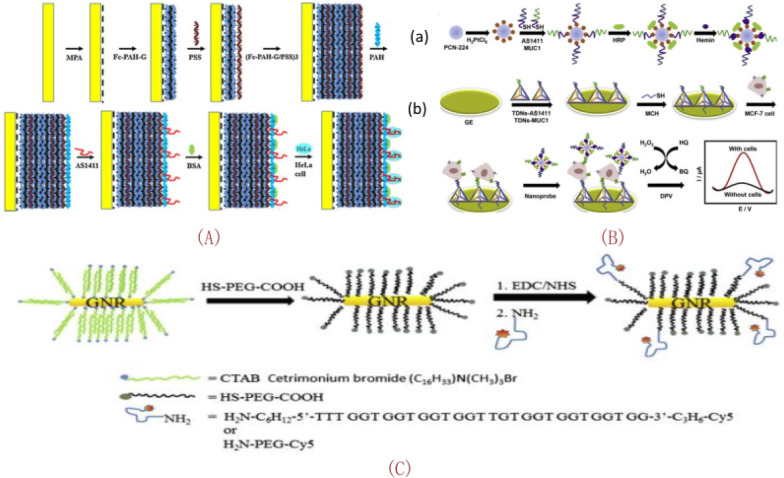
**A** Schematic diagram of the construction process of biosensor LBL technology for detection of HeLa cells. **B**(a) Preparation process of PCN-224-Pt/HRP/ Dual-Aptamer /GQH nanoprobe. (b) Schematic diagram of the manufacturing process of the electrochemical biaptamer cell sensor. **C** Schematic diagram of GNRS binding of synthetic fluorescent aptamers

As one of the most basic materials of life, nucleic acid plays an extremely important role in life activities such as genetic information storage and protein synthesis. Nucleic acid includes RNA and DNA. Studies have shown that many genetic diseases and cancers are related to genetic abnormalities [233]. For example, microRNAs (miRNAs), a widely studied class of non-coding single-stranded RNAs, are abnormal in a variety of cancer cells. Therefore, detection and analysis of specific sequence nucleic acids can provide important reference information for clinical diagnosis, pathogenesis research, and gene therapy. Molecular probe is a special kind of drug preparation, which connects medical imaging equipment (such as CT, MR1, ultrasound and nuclear medicine) with disease characteristic molecules [13, 164, 234–236]. Ma et al. [160] reported that a fluorescent probe, Cy3, was anchored to the terminal of the targeted aptamer AS1411 of glioma cells to form Cy3-AS1411, which was then conjugated with BBB-TGN of a targeted peptide 30 via a polyethylene glycol conjugate. Wang et al. [237] reported the construction of a novel unlabeled aptler for detection of cancer cells by layer by layer assembly (LBL) of FC-PAH-G, PSS and aptler AS1411. The aptamer sensor has the characteristics of high sensitivity and good stability. As shown in Fig. 14A, the scheme of making biosensor using LBL technology is shown. Among them, LBL technology provides a more effective probe for the preparation of aptamer sensor, thus amplifying the signal and improving the sensitivity of detection. Based on the insulating property of cell membrane, the current response of sensing electrode will be reduced when cancer cells combine with the G-quarkazin structure formed by AS1411, which reflects the sensitivity of HeLa cells to the change of current signal. An electrochemical aptamer cell sensor for highly selective detection of MCF-7 cells was constructed in 2019 [238] based on dual-recognition aptamer and nanoscale electrocatalyst layering (LBL) technology for signal amplification. The cell sensor consists of two parts: a nucleic acid modified gold electrode connected by two aptamers and a multifunctional hybrid nano-probe. As shown in Fig. 14B(a), the use of newly introduced synthetic nanostructures as nanocarriers increases the amount of biocatalyst. Pt nanoparticles were modified on the surface of PCN-224 nanomaterials due to electrostatic attraction, and then GQH deoxyribozyme, horseradish peroxidase and bisaptamers were fixed on the surface of PCN-224-platinum nanoparticles, thus forming an electrochemical hybrid nano-probe. As shown in Fig. 14B(b), phenol-224-platinum, horseradish peroxidase and GQH deoxyribozyme were used as catalysts for signal amplification on the cell sensor based on the electrochemical properties of benzoquinone generated from the oxidation of HQ by hydrogen peroxide. The number of electrochemical nanoprobes on GE is closely related to the signal of the electrochemical cell sensor. As shown in Fig. 14C, the Maria Elena Gallina group [167] catalyzed by EDC/NHS, carried out ligand exchange, and then bio-coupled with anticancer oligonucleotide AS1411 to generate fluorescent, biocompatible, Aptamer coupled GNRs. H_2_N-AS1411-Cy5 was conjugated by modification. It can be used as a selective multifunctional probe for cancer in photothermal therapy.

#### Protein detection

**Fig. 15:**
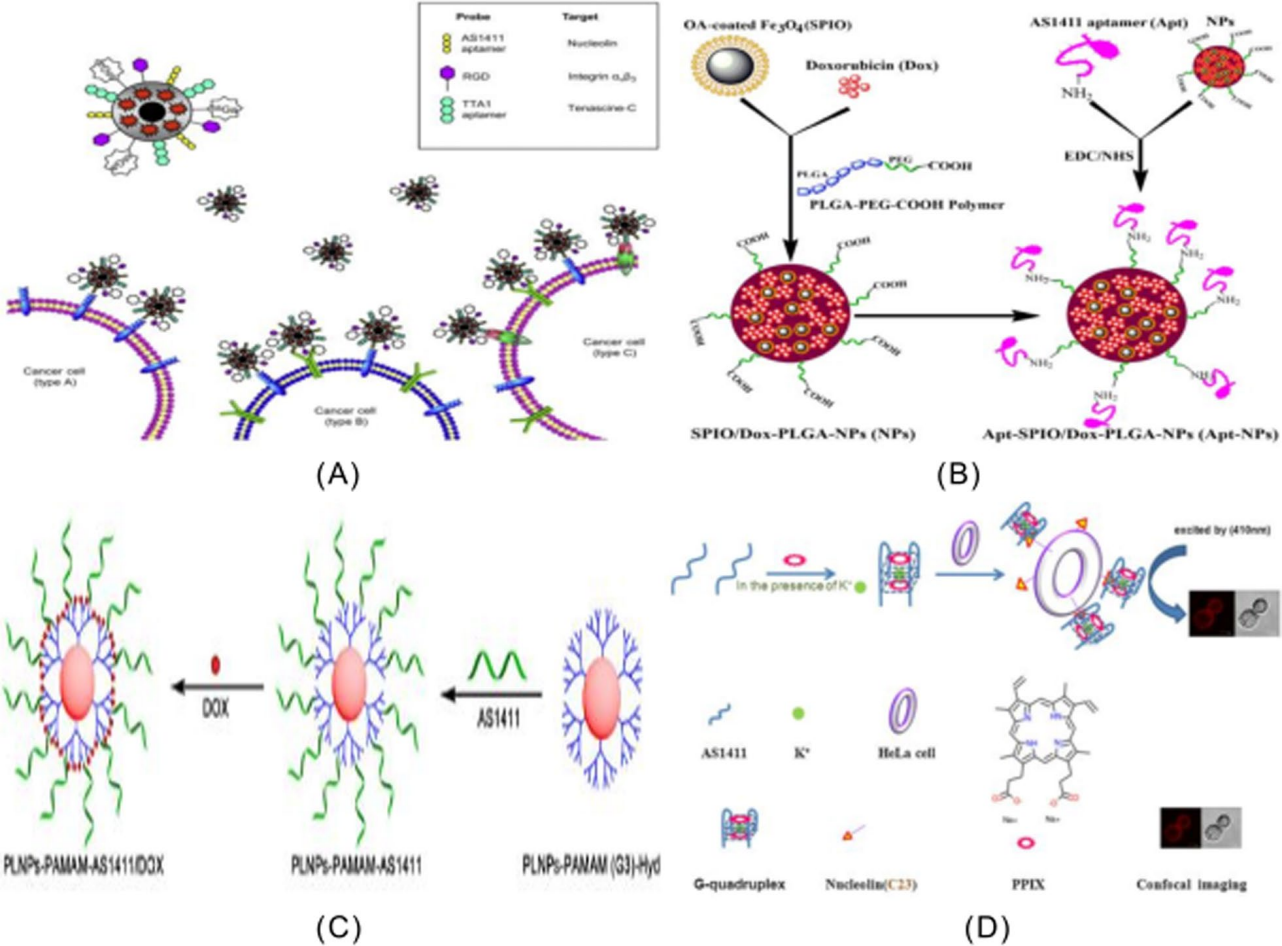
**A** Strategies for targeting various cancers using SMART cancer probes based on multi-peak nanoparticles. **B** Schematic diagram representing APT-NPS formula. **C** Scheme for the synthesis of PLNPS-PAMAM-AS1411/DOX. **D** Schematic of biological imaging of cancer cells with the AS1411-PPIX complex

Protein plays an important role in living organisms. Some proteins, such as mucin (MUC1), nucleolus protein and carcinoembryonic antigen (CEA), are overexpressed after cells become cancerous. These proteins can be used as protein tumor markers [239]. Therefore, the development of sensitive detection methods for the early diagnosis and treatment of cancer has important guiding significance. For tumor-associated proteins, DNA sequences of specific binding targets were collected from a huge DNA library containing 10^13^–10^16^ random sequences [207, 240, 241]. With the development of tumor molecular biology research, cancer tissues express different components from normal tissues at an early stage, such as some tumor special groups, special proteins, etc. With the deepening of research on tumor molecular biology, cancer tissues express components different from normal tissues at an early stage, such as some special tumor groups and special proteins [70, 242, 243]. The Maria Elena Gallina group [167] synthesized fluorescently, biocompatible and high-purity GNRs with clear aspect ratio through bio-coupling with anticancer oligonucleotide AS1411 after exchanging ligand. This GNRs is an ideal multifunctional selective probe for cancer. For use in breast cancer cell imaging and targeted drug delivery. A dual-targeted DNA tetrahedral nanocarrier (MUC1-TD-AS1411) system was developed in 2018 [244] to effectively improve the efficacy of chemotherapy and achieve real-time imaging of tumor cells. The nanocarrier consists of DNA tetrahedral core, MUC1-probe and AS1411 aptamer. The DNA tetrahedral core of the first part is mainly used for polyvalent binding of functional ligands and Dox load. The second part of the MUC1-probe is the activated MUC1 aptamer probe, which is mainly used to target the MUC1 protein on the cell membrane for imaging. The probe is formed by hybridization of the MUC1 aptamer sequence with a fluorophore extending from a vertex and a sequence complementary to the quenching agent. The third part, AS1411, is used to bind nucleoproteins and is formed by hybridization of elongated sequences with protruding ends on three vertices. Hae YoungKo et al. developed and designed a targeted (SMART) cancer imaging probe based on the simultaneous multiaptamer and RGD multipeak nanoparticles produced by AS1411 and TTA1 aptamers and arginine-glycine aspartate (RGD) peptide. As shown in Fig. 15A, SMART cancer probe consists of AS1411 aptamer targeting nuclear protein, TTA1 aptamer targeting Tnc protein, and RGD peptide targeting integrin α_V_β_3_, binding to multipeak nanoparticles capable of simultaneous acquisition of fluorescence, radioisotope, and magnetic resonance images. Three different cancer probes were simultaneously bound to MF nanoparticles, and the SMART cancer probe was designed with the MF/AS1411/TTA1/RGD molar ratio of 1:1:1:1 without considering the binding order [245]. Based on the targeting effect of AS1411 aptamer (Apt) in super paramagnetic iron oxide nanoparticles (SPIONs)/Dox co-deposited poly-lactic-glycolic acid (PLGA) nanoparticles on mouse C26 colon cancer cells. Jafar Mosafer group [246] developed multiple emulsion solvent evaporation for the treatment of C26 colon cancer. As shown in Fig. 15B, the schematic diagram of covalent binding between carboxylate functionalized nanoparticles and Apt-NH_2_ molecule is presented. Among them, nucleolin plays a role as a targeting ligand that can promote the anti-tumor delivery of AS1411 targeted NPs. The formation of Apt-NPs can not only accelerate the cell uptake rate of Dox by C26 cancer cells, but also increase the cytotoxic effect of Dox. Because APT-NPs has dual therapeutic and diagnostic functions in cancer, Apt-NPs can be considered as a good tumor targeted drug delivery system. Zhang et al. [247] reported a preparation scheme for a compound PLNPs-PAMAM grafted with a terminal hydrazine group as a nanomaterial for aptam-directed imaging and acid-responsive drug delivery. As shown in Fig. 15C, the core of the nanoplatform is composed of PLNPs because the ultra-long near-infrared can emit light continuously; In order to improve the biocompatibility of the nanoplatform, polyamide-amine was grafted onto the surface of PLA nanoparticles. The aptamer AS1411 was bonded to the surface of PLNPS-PAMAM (G3) by an amide condensation reaction. The role of aptamer AS1411 is to endow the nanoplatform with a highly specific targeting ability to nucleolin overexpressed on the cancer cell membrane. Ai et al. [248] reported a new method for in-situ labeling and imaging of protein nucleoproteins in HeLa cancer cells using a multifunctional anticancer aptamer (AS1411) and its fluorescent ligand protoporphyrin (PPIX). As shown in Fig. 15D, in the presence of potassium ions, AS1411 can fold into a G-quadruplex structure and bind fluorescently enhanced fluorescent ligand protoporphyrin (PPIX) to target the over-expressed nuclear proteins of cancer cells. At this time, specific biological imaging of cancer cells can be realized by laser scanning confocal microscope. Biologic imaging of the AS1411-PPIX complex produced by binding can significantly distinguish HeLa cancer cells from normal cells.

## The latest research on AS1411

Although AS1411 has been withdrawn from clinical trials at present, the development of AS1411 nanomaterial complexes as targeted molecules can make significant contributions to the research related to cancer treatment, so the research on its structural optimization and the role of its nanomaterial is still very hot [176, 249–257]. The following new studies of AS1411 have been reported in recent 6 months, which proves that although AS1411 has been developed for many years, the functional development of its nanocomposites is still a hot research issue at the present stage.

**Fig. 16:**
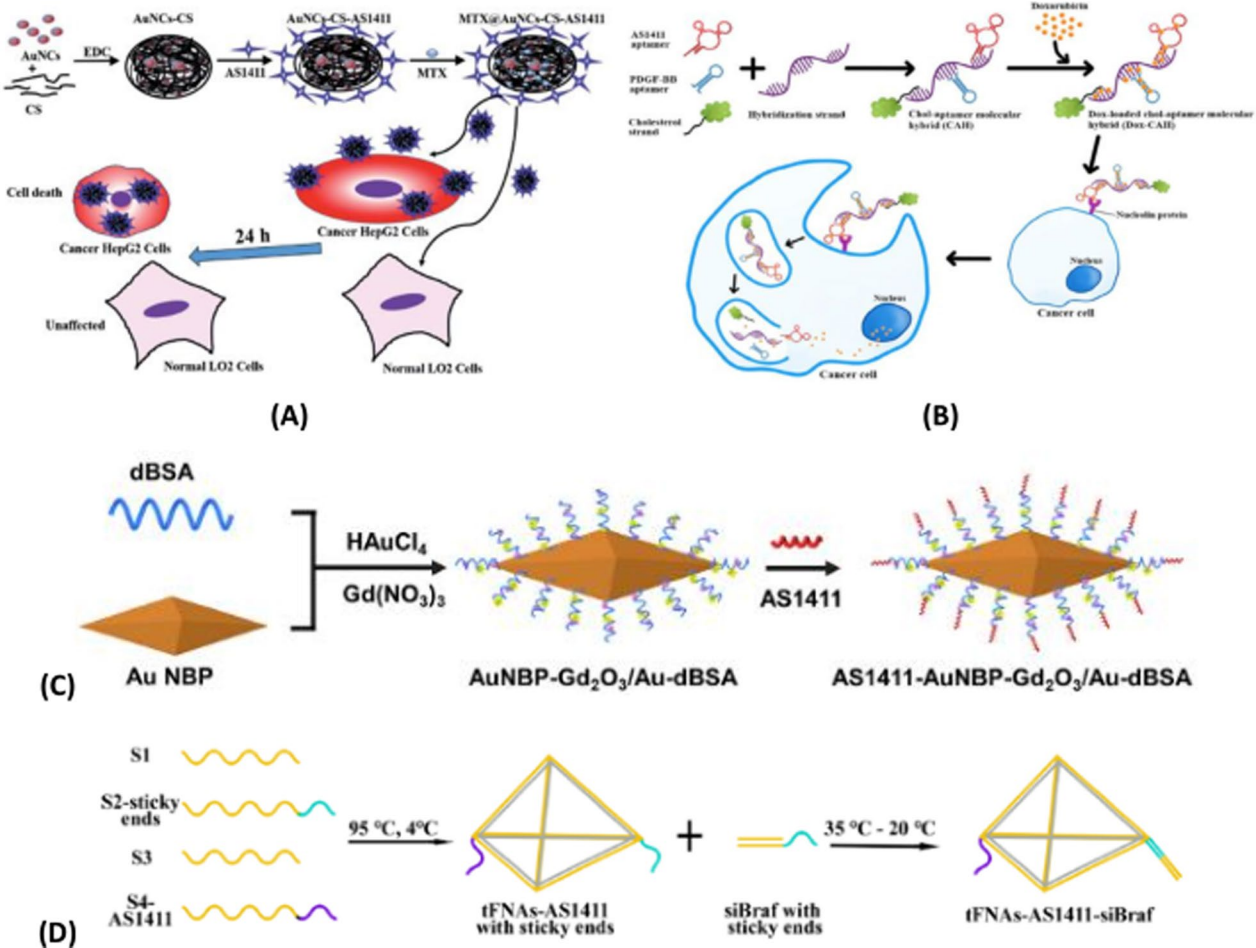
**A** MTX@AuNCs-CS-AS1411 synthesis and targeted drug delivery diagram. **B** Synthesis diagram of Dox-Chol-aptamer molecular hybrid (CAH). **C** Schematic illustration of synthesis of AS1411-AuNBP-Gd_2_O_3_/Au-dBSA nanocomposites. **D** The construction of tFNAs-AS1411-siBraf system

In 2021, Zhang Group [258] Synthesized AuNCs-conjugate chitosan (CS) nanocarriers (AuNCS-Cs) by covalent reaction, and then conjugated with anti-nucleoside aptamer (AS1411) to synthesize chitosan based nanocarriers capable of cancer cell imaging and targeted drug delivery. The principle of action is mainly a hydrophobic model anticancer drug, methotrexate (MTX) is wrapped into tumor-targeted nanocellular carrier through hydrophobic interaction. The nanocervator(MTX@AuNCs-CS-AS1411)was then used for site-specific detection and targeting of human hepatocellular carcinoma cell line HepG2 overexpressing nucleolar protein receptor. The specific process is shown in Fig. 16A. Pichayanoot Rotkrua group [259] developed a Dox-loaded Chol–aptamer molecular hybrid (Dox-CAH) loaded with doxorubicin, cholesterol and nucleolar protein and platelets-derived growth factor BB (PDGF-BB), as shown in Fig. 16B. CAH showed specific binding to SW480 due to the presence of AS1411 aptamer. Wang et al. [260] developed a nanosystem for FL/MR dual-mode image-guided photothermal therapy using Au nanoclusters and denatured bovine serum albumin(AuNBP-Gd_2_O_3_/Au-dBSA) in combination with AS1411 to ensure targeting of breast cancer cells in Fig. 16C, The main function of AS1411 is its own targeting. Since most malignant melanoma is related to Braf gene mutation, the Xiao group [261] used Tetrahedral framework nucleutics (tFNAs) synthesized from four single-stranded DNA to modify the target genes below Braf siRNA (siBraf). tFNAs-AS1411-siBraf system was constructed by integrating AS1411 adaptor. Among them, the role of AS1411 is to significantly improve the uptake efficiency of cells. As shown in the Fig. 16D, because tFNA is synthesized by four single-stranded DNA (ssDNA), two vertices of tFNA are modified by AS1411 and siBraf, respectively. AS1411 is directly connected to the 5'end of S4 (S4-AS1411), and after synthesis of tFNAs-AS1411, siBraf is connected to it through the viscous end. Since S2 and siBraf have sticky ends, the sticky ends are modified to be the 3'ends of S2 and the antisense chain of siBraf.

**Fig. 17:**
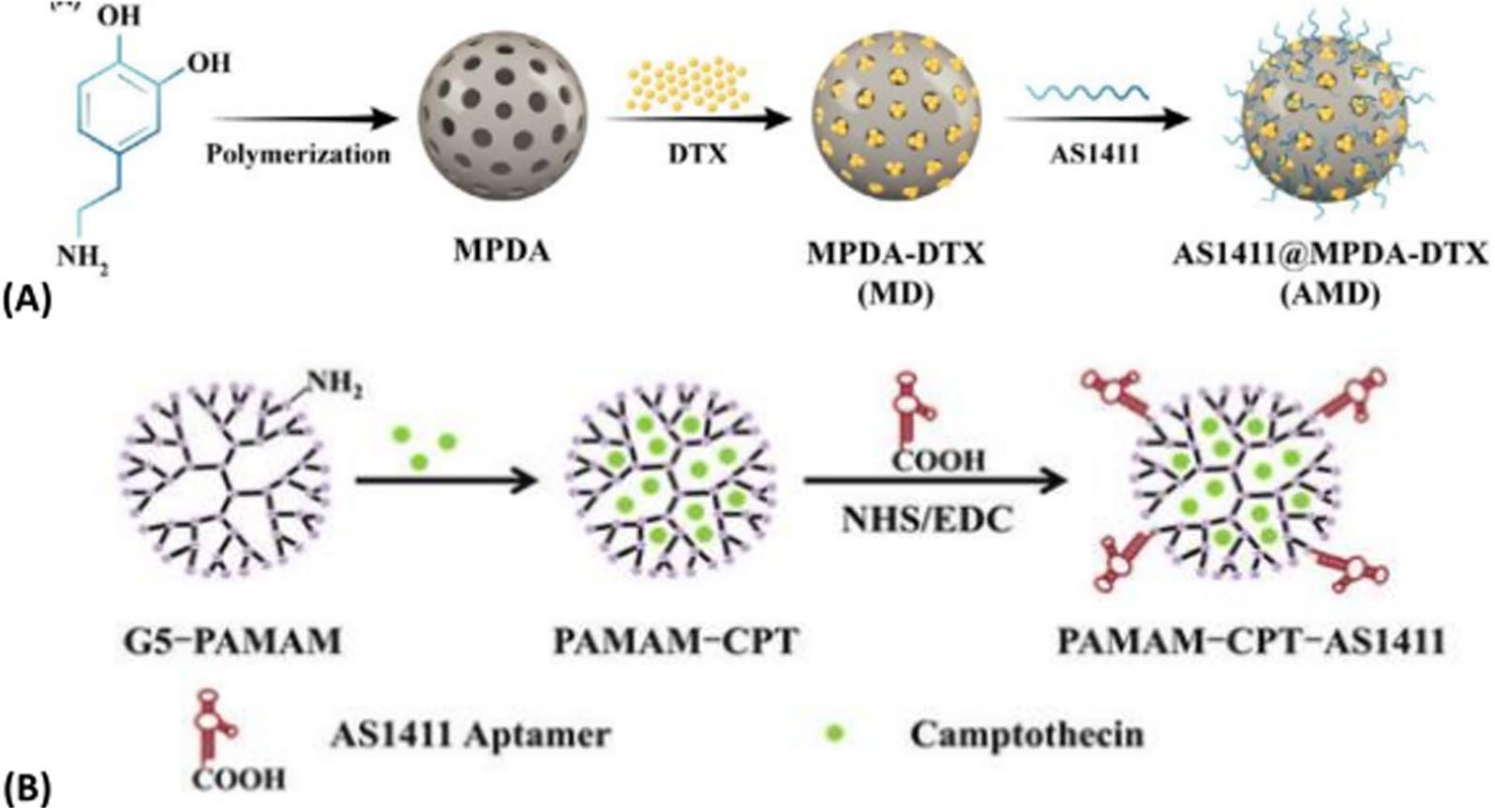
**A** AMD synthesis diagram. **B** Synthesis diagram of PAMAM-CPT-AS1411

There are also emerging uses for targeted delivery in combination with the Chinese herb extract paclitaxel. Wang et al. [262] used a novel microemulsion consisting of two tumor- targeting ligand (T7 peptide and AS1411 aptamer), ultra-small superparamagnetic iron oxide nanoparticles (Fe_3_O_4_), and shikonin&docetaxel-coloaded microemulsion (SKN and DTX-M) of shikonin and docetaxel system (Fe_3_O_4_@T7/AS1411/DTX&SKN-M) for targeted delivery of glioma. This system can not only circulate stably in the blood, but also distribute evenly in glioma under the external magnetic field through the affinity between nucleolins and transferrin receptors, which is used to prevent the growth of glioma in situ. Moreover, Fe_3_O_4_ nanoparticles are superparamagnetic because they are wrapped in the core. This system penetrates BBB via T7 peptide and enters glioma cells with the assistance of T7 peptide and AS1411 aptamer. Dei group [263] incubated mesoporous polydopamine (MPDA) nanoparticles loaded with DTX, and modified AS1411 aptamer into MPDA, as shown in Fig. 17A AS1411@MPDA-DTX (AMD) system is constructed by using covalent reaction. The nanocomposites can effectively target prostate cancer cells under acidic conditions, promote the internalization of DTX, and improve the efficiency of anti-prostate cancer. Liu et al. [264] constructed PAMAM-CPT-AS1411 nano drug carrier using the high sensitivity of atomic force microscope (AFM) tip cantilever beam. It is used to screen highly effective nanomedicine. The synthetic composition is shown in Fig. 17B, where CPT is encapsulated in the lumen of G5-PAMAM and AS1411 is covalentally conjugated with PAMAM-CPT surface.

## Summary

Cancer has become one of the main reasons threatening human health for nearly a century due to its strong growth ability and uncontrolled characteristics, making early diagnosis especially important. With the improvement of traditional methods, a convenient design and specific targeting method has been developed for the detection of tumor markers. The tumor markers discussed in this paper mainly include miRNA, glycosylase and nucleolin.

With the development of nanotechnology, many nanomaterials that can be used as signal reporting and delivery carriers have been introduced. Based on the uniqueness of nucleic acid nanomaterials, AS1411 aptamer was used as the targeting agent in this review to combine with various new nanomaterials. The nanomaterials discussed include graphene nanomaterials, mesoporous silica nanomaterials, silver nanomaterials, gold nanomaterials and other nanomaterials. Different types of nucleic acid functionalized nanomaterials with different functions can be generated by different methods. These functionalized nanomaterials can not only recognize the key structures expressed in cancer cells and their surrounding tissues for drug delivery, but also act as direct cancer therapeutics themselves. Active or passive targeting of tumor markers, and highlights recent research in biosensors, biomedical, drug targeted delivery, and nanoprobe imaging using the AS1411 aptamer nanomaterial hybrid platform. The specific functional classification of AS1411 is shown in Table 2.

**Table 2:** AS1411 function table

	Principle	Role	Refs
AS1411	Inhibition	By inhibiting the binding of nucleolin to mRNA, the mRNA that encodes the anti-apoptotic regulator Bcl-2 was destabilized	[91]
		It can bind to the nuclear protein on the surface of cancer cells and inhibit the NF-κB pathway	[121]
		It acts by inducing cell cycle arrest, inhibiting DNA replication and inducing apoptosis	[90]
		AS1411 was combined with polyethylene glycolated cationic liposomes as a targeting probe ASLP. ASLP has inhibitory and cytotoxic effects on melanoma cells	[195]
		DOX-Apts-CS-AuNPs can selectively carry their therapeutic agents to cancer cells, thereby reducing the systemic effect of DOX and significantly inhibiting tumor growth	[116]
		For nucleoprotein inhibition, the combination of aptamer AS1411 or nucleosome NCL with doxorubicin reduces the survival rate of DLBCL cells	[191]
		HA@APT§DOX is designed for deep tumor suppression. It can effectively enhance the accumulation of drugs in tumor sites and inhibit tumor growth. DOX is released from APT in an acidic environment and transported to the nucleus to achieve the drug push for deep tumor inhibition	[145]
		AS1411 was mercapto conjugated to the surface of AUND to enhance its intracellular uptake and tumor-targeting capability, which was used to enhance its intracellular uptake by human breast tumor MCF-7 cells	[114]
		AS1411NTRs co-loading with DOX and TBO significantly inhibited the growth of MCF-7/ ADR cells, and DOX was mainly located in the nucleus. Targeting AS1411NTRs and DOX + TBO co-loading can help overcome resistance. The drug delivery system enhances its in vitro cytotoxicity and overcomes drug resistance in DOX-resistant human breast cancer cells (MCF-7/ ADR)	[197]
		NiCo-PBA@Tb^3+^@PEGMA functionalization with AS1411 enables rapid drug release within cancer cells and enhances tumor-targeted delivery of doxorubicin. NiCo-PBA@Tb^3+^ @ap@dox is abundant in tumor tissue. It can effectively inhibit tumor growth and show enhanced anticancer activity in vivo	[188]
		AS1411-TDN is a more powerful tumor targeting vector, and AS1411-TDN itself can inhibit tumor cell proliferation, thus enhancing the anti-tumor effect	[107]
		Binding with nucleoprotein leads to Rac1 continued activation and non-apoptotic cell death, a metastasis-inhibiting mechanism. Cancer cells may overexpress nucleoprotein to overcome this barrier	[81]
		NCL selectively mediates the binding and uptake of AS1411. It is used to effectively inhibit the attachment/entry of human immunodeficiency virus type 1 (HIV-1) into host cells	[91]
		A polyelectrolyte complex of TD-miR-chitosan was modified with antinucleolin aptamer AS1411 to act as an intelligent gene delivery system for storage, transfection and release of goods into the cytoplasm. Used to suppress breast cancer, dual targeting reduces the drug dose and side effects on normal cells	[190]
		AS1411 inhibited proliferation, migration, tube formation and expression of miR-21 and miR221 in HLSC stimulated by recombinant human vascular endothelial growth factor. To inhibit corneal neovascularization	[83]
		AS1411 was conjugated to the surface of PLNPS-PAMAM to enhance the intracellular accumulation of nanoparticles.PLNPS-PAMAM-AS1411 /DOX for precision cancer therapy	[246]
		DNCA lipids can induce the formation of secondary structure of AS1411 G4 and release oligonucleotides in cells. DNCA/AS1411 nanoparticles showed good biocompatibility in vitro and in vivo. Penetrate into cancer cells and promote apoptosis leading to potent anticancer activity, especially against paclitaxel-resistant cells	[141]
		Phase II clinical trials for acute myeloid leukemia and renal cell carcinoma. The growth of gastric cancer cell lines was inhibited in a dose-dependent manner	[137]
AS1411	Competition in the role	AS1411 binding nucleoli has high affinity and is considered as a molecular decoy that competes with Bcl-2 binding nucleoli	[123]
		AS1411 initiates the hybridization of acid-modified oligonucleotides to form hydrogels, and the presence of the target protein nucleoprotein leads to the dissolution of the gel, resulting in a reduction in cross-linking density through competitive target aptamer binding. Drug carriers for targeted therapy and other biotech applications	[192]
		AS1411, TTA1 and RGD probes combined with MF nanoparticles for nanoparticle cancer imaging probes	[244]
	Targeted role	Combine with quantum dots and use them in combination with drugs that are inserted into DNA for simultaneous targeting, imaging and treatment of cancer	[62]
		Bindingnucleoli, a protein highly expressed in the plasma membrane of cancer cells, has been successfully used as a targeted ligand to track C6 glioma cells	[89]
		Conjugated with MF nanoparticles for visualizing the location of nucleolar proteins or miRNA biogenesis in vitro and in vivo	[243]
		5-FU as a new tumor-targeting nanomedicine (AS1411-T-5-FU) was attached to the DNA-based delivery system to enhance the therapeutic efficacy and targeting of breast cancer cells for targeting to kill breast cancer cells	[151]
		APT-Dox-PLGA-PVP nanoparticles are released by nucleolin receptors to enhance the therapeutic efficiency. A novel drug delivery system for enhanced anti-lung cancer therapy	[90]
		DOX hydrochloride inserted into aper AS1411 (AP-DOX) is encapsulated in the aqueous interior of liposome (LIP (AP-DOX)) and enters the nucleus after strong binding with nucleolin. By using this drug delivery system, DOX hydrochloride can be effectively accumulated in the nucleus to effectively kill breast cancer cells	[94]
		Identifying C6 cells and mediating endocytosis greatly increased the intake of ASNP and ASTNP by C6 cells. Modulate the uptake of nanoparticles by endothelial cells and cancer cells	[191]
		A three-strand linked pocket DNA nanostructure composed of three AS1411 apers for the delivery of doxorubicin (Dox) to cancer cells	[120]
		AS1411 aptamer was further modified to MSNs for cell/tumor targeting. For in vitro and in vivo treatment of triple cancer	[107]
		The NAANPs formed can be used to differentiate cancer cells from normal cells by targeting the cell surface through specific AS1411-nucleolin interactions	[122]
		AS1411-conjugated NP enhances cell proliferation inhibition due to the selective delivery of GEM to nucleolin-overexpressed cancer cells. For the treatment of non-small cell lung cancer by targeting gemcitabine	[84]
		A system of AS1411 aptamers (for glioma targeting) and TGN peptides (for BBB targeting) modified nanoparticles (ASTNPs) is designed to target brain gliomas and improve the survival rate of glioma carrying mice	[191]
		Active targeting of AS1411 liposomes significantly increases the accumulation of DOX in tumor tissues, inhibits tumor growth and reduces systemic side effects, and is used in therapies to overcome the effects of multidrug resistance	[230]
		DOX loading and micellar stability were improved by a binary hybrid system consisting of AS1411 modified Pluronic F127 and β-cyclodextrin linked polyethylene glycol-B12 polylactic acid.Used to demonstrate prolonged circulation time, increased antitumor activity, and reduced cardiotoxicity	[11]
		The ability of AS1411 aptamer and MUC 1 aptamer to bind to cancer cells is used in multiple analysis and multicolor imaging of tumor cells. Especially when it comes to selecting aptamers that are more targeted	[109]
		A modified and promoted dendritic macromolecule was designed by MUC1, AS1411 and ATP aptamers for targeted delivery of EPI and evaluated for efficacy in target cells including MCF-7 cells (breast cancer cells) and C26 cells (mouse colon cancer cells)	[192]
		The gold nanocluster (AuNC), the ring-shaped RGD (CrGD) of integrin and the aptamer AS1411 (APT) were combined. A novel nanoplatform with dual targeting function (AuNC-CrGDAPT) was established	[114]
AS1411	Targeted role	The DNA of AS1411-modified gold nanoparticles and DOX-rich gas chromatographic inserts was used for the release of DOX from hairpin structures. Shows significant targeted binding in the treatment of SW480 colon cancer cells	[115]
		Novel drug delivery systems for functionalized nanocapsules have been developed for active drug targeting and are good candidates for drug delivery systems for cancer therapy through poly(n-vinyl pyrrolidone-alt-itaconic anhydride) and aptam-functionalized chitosan	[192]
		The toxicity of C8 in cultured melanoma cells was increased by AS1411-ICG-C8. It also reduced the tumor growth of mouse melanoma induced by the B16 cell line in vivo. For effective removal from the mouse body via the kidney	[144]
		AS1411 aptamer functionalized albumin nanoparticles loaded on iron oxide and gold nanoparticles for targeted delivery of the anticancer drug doxorubicin (DOX)	[244]
		AS1411 aptamer is bound to the surface of a matrix metalloproteinase-2 reactive polymer preparation to provide directed drug delivery to nucleolin-positive cells. It is used to treat colorectal cancer	[143]
		SiNP -DOX-AAPaT has significant apoptotic effect and anti-tumor properties on cancer cells. SiNP-DOX-AAATP provides an intelligent delivery system for controlling the encapsulation and release of DOX at the disease site	[106]
	Cancer detection	Cells were effectively captured on the electrode surface by the specific binding between the cell surface nucleoprotein and aptamer AS1411	[124]
		The expression of nucleolin in hepatocellular carcinoma (HCC) cell lines and HCC tissues was determined	[134]
		Binding with nucleolin greatly enhanced the fluorescence intensity of silver nanoclusters. Direct biological imaging of HeLa cells	[110]
		Biometric imaging of the AS1411-PPIX complex can clearly distinguish HeLa cancer cells from normal cells and can be achieved in human serum	[247]
		A simple and ultra-sensitive fluorescence aptamer for Cu^2+^ detection was prepared by using AS1411 aptamer as a targeting agent, its complementary chain and streptomavidin-coated magnetic beads.Used to measure serum levels of Cu^2+^ in patients with Wilson's disease, Alzheimer's disease and diabetes	[93]
		Cytotoxicity and uptake of the tested HGN-AS1411 nanoconjugate by A375 and HaCaT cells were assessed.Used for skin cancer treatment	[195]
		AS1411 was used to construct a ratio pH biosensor by covalently modifying unlabeled fluorescent silicon nanocrytes with a crosslinker. Lysosomal imaging and pH measurement of MCF-7 cells	[141]
		This aptamer antiproliferative effect results from binding to nucleolus proteins localized in the nucleoli that can be transferred to the surface of certain cancer cells	[150]
		CdTe quantum dots coated uniformly on the surface of MSNs were coupled to construct a nanobiological probe to selectively collect and detect breast cancer cells MCF-7	[107]
		A polydimethylsiloxane (PDMS) based microfluidic platform for the detection of NCl using aptamer AS1411-N5	[94]
		The vector is in situ functionalized by the nucleolin apperer AS1411 to improve the delivery and uptake of the complex to target cells	[211]
		Tetrahedral DNA nanostructured biaptamers (AS1411 and MUC1) are immobilized on the surface of the gold electrode as biometric elements to capture MCF-7 cancer cells and improve the density and orientation of the surface nanoprobes. For highly selective detection of MCF-7 cells	[74]
		The AS1411 aptamer without any functionalization was immobilized on the copper nanoparticle coating and G-quadruplex aptamer was trapped on the metal surface to facilitate cancer diagnosis	[149]
		The binding affinity of AS1411 for nucleoprotein and the high fluorescence of silver nanoclusters provide an opportunity for its application in intracellular imaging and nuclear staining. To detect enhanced photodynamic efficiency of colon cancer cells	[166]
		A novel topical gel formulation based on sodium alginate and hyaluronic acid contains AS1411 aptamer-functionalized polymer nanocapsules loaded with an anti-tumor agent (5-fluorouracil) for the treatment of skin cancer	[5]
AS1411	Cancer detection	The sensing mechanism of PGO-I^−^-ISE cells was demonstrated by the I^−^sensing curves that were parallel transported with different concentrations of cell solutions after incubation.Used to detect selectivity to target cancer cell HeLa in the blood environment	[59]
		TGN is conjugated with Cy3-AS1411 via a polyethylene glycol connector and is ingested through endothelial monolayers and glioma spheres. It is used to target glioma cells and endothelial cells	[159]
		Cu-CB-TE2A-AS1411 was used for aptamer imaging to detect lung cancer	[87]
		The aptome AS1411 provides a good choice for NCL recognition without penetration because it has better cell uptake and enhanced stability for visualizing the distribution of NCL	[198]
		DOX @APT-PEG-PDA-Molybdenum Disulfide Nanoparticle shows that this Molybdenum Disulf-based drug delivery platform is promising for targeted synergistic therapy of cancer	[126]
		PSF/GO-TAPP/rGO-FET biological conjugate was applied to trace detection of CTCs and differentiation of tumor cells and white blood cells under the catalysis of platinum nanoparticles, showing high specificity and sensitivity	[99]
		An unlabeled aptamer sensor composed of PAH-glucose, sodium alginate diester, and aptamer AS1411 for detection and identification of cancer cells	[98]
		The aptamer AS1411 and amphoteric peptide were simultaneously immobilized by polydopamine (PDA) ligation.6- mercaptohexanol as the blocking molecule.Used for quantitative detection of target cells	[126]
		The interaction between nucleolin and AS1411 causes a change in surface stress, resulting in a differential deflection between the sensor and the reference cantilever. For the unlabeled detection of nucleolins	[93]
		Bioconjugation with anticancer oligonucleotide AS1411 resulted in fluorescent, biocompatible, appers-conjugated GNRs. For selectively targeting cancer cells	[166]
		The specific binding of AS1411 to target cells triggered the aptamer removal from the solution. The hybridization-based method showed a selective colorimetric response detectable to the naked eye in MCF-7 cells	[118]
		AS1411 aptamer binds to camptothecin loaded PEGylated dendrimer surfaces, providing site-specific delivery of camptothecin, inhibiting C26 tumor growth in vivo and significantly reducing systemic toxicity. It is used to treat colorectal cancer	[214]
		Prolonged retention of AS1411-polyethylene glycol-manganese dioxide nanoprobe in 786–0 renal carcinoma tumors in vivo. It is eventually excreted from the body by a renal clearance route. It was used to realize the specific magnetic resonance imaging of mice with renal carcinoma, and significantly accumulated in the tumor and prolonged the retention time	[139]
		After cDNA and AS1411-Rox were fixed on the surface of the particles, NCL was transported through an electric-field-driven nanoparticle channel, and As1411-Rox was removed through a specific recognition interaction for the ultra-sensitive in-situ detection of NCL	[96]
	Identification of the nature	Coated with crude protein, APT-Fe-AuNPs showed good cytotoxicity to human lung cancer cells	[240]
		Phase II clinical trials were conducted as anticancer agents. Shows extremely effective antiviral activity and is used in new anti-HIV drugs	[138]
		AS1411 was incorporated into DOX-loaded BSA surfaces, and specific recognition between receptors overexpressed on tumor cells, aptam-modified nanoparticles DOX@Apt-BSA showed enhanced cell uptake and increased cytotoxicity	[194]
		AS1411NTRS nanochains are used to study the interaction between DOX, EPI or DAU and nanochains. Three anthracycline antibiotics were released from AS1411NTRs.Used for testing cytotoxicity of target and non-target cells	[197]
		CTCs were identified and isolated from whole blood samples by magnetic nanoparticles modified with CTCs-specific aptamer AS1411	[125]
		This leads to increased accumulation of nanostructures in nucleoprotein positive cancer cells through active targeting. BSA binds to colorectal cancer cells through REDOX—prone disulfide bonds and has shown significant therapeutic efficacy in cancer cells	[76]
		D-/ L-isoT and 20-DI were added to enhance the bioactivity of AS1411, and animal experiments were conducted to evaluate its antitumor effect in vivo	[44]
AS1411	Common dosing	DNPs Clolar/AS1411 / HA/RNA/DOX multifunctional nanoparticles, improve the tumor site drug accumulation, eventually synergy inhibit tumor growth through a variety of drugs	[145]
		Polyethylene glycolated rod-like mesoporous silica nanoparticles prepared with camptothecin loading apt-peg@msnr-Cpt /Sur labeling to provide selective treatment for colorectal adenocarcinoma were used in the AS1411 DNA apposer labeling system to promote drug uptake in nucleolin-positive colorectal cancer cells	[126]
		TMPyp&DNM &Apt-gc34@SPION NPS with pH-dependent conformational changes in the I-sequence establishes a single system of dual-targeting, dual-therapeutic and pH-stimulation-responsive drug release.Used for pH-controlled release of DNM in the microenvironment of cancer cells	[90]
		AS1411 /SKN&DTX-M was selectively accumulated in the brain of in situ luciferase transfected nude mice with U87 glioma. Co-delivery of hyaluronic acid and AS1411 aptamer bifunctionalized shikonin and docetaxel for combinational antitumor therapy with dual agents	[146]
		The AS1411-modified LDL responds to surrounding pH conditions and is used to selectively release anti-tumor drugs to cancer cells, reducing the systemic effects of DOX	[39]
		HeLa and non-malignant cells were cytotoxic, and when combined with AS1411 derivatives, the cytotoxicity of the free ligand to non-malignant cells was inhibited. It can dissociate intracellular to deliver C8T to the nucleoli. AS1411-derived G4S can be used as a potential cancer drug delivery system for cervical cancer	[64]
	Cells capture	By binding to nuclear proteins that are overexpressed on the surface of the cancer cell membrane. The resulting titanium dioxide nanofiber substrates exhibit specificity through synergistic topographic interactions. TiO_2_-BSA-Biotin -AS1411 specifically captures RARE CTCs	[128]
		AS1411 was treated with gold nanoparticles modified with secondary aptamers. Sensitive ECL detection for MCF-7 cancer cells. In order to specifically capture cancer cells, the anode of gold BPE is modified with aptamers	[124]

Finally, AS1411 aptamer nanomaterials are widely used in the detection of cancer cells such as lung cancer, brain cancer, cervical cancer, etc., but there are still some difficult problems to overcome: (1) AS1411 aptamer nanomaterials are not degradable in vivo, and the safety of drug delivery system is still to be studied; (2) The shape and size of AS1411 aptamer nanomaterials are related to the drug administration effect. The preparation of AS1411 aptamer-nanomaterials with the best effect should be further studied; (3) The targeting effect should be further improved. In order to solve these problems, better combination of AS1411 aptamer and nanomaterials is needed, and further development in the enrichment of AS1411 aptamer and nanomaterials is needed in the future. So far, studies on AS1411 have been applied in various aspects. Although it is not new in clinical trials, the future of AS1411 is far more than that. In the latest research, using AS1411 to modify nanoparticles loaded with drug active extracts has once again become a hot topic. AS1411 will still play an irreplaceable role and research significance in cancer treatment.

## Data Availability

All data and materials in this study are included in the published article and its additional file.
